# Hepatocyte-specific METTL3 ablation by Alb-iCre mice (GPT), but not by Alb-Cre mice (JAX), resulted in acute liver failure (ALF) and postnatal lethality

**DOI:** 10.18632/aging.205753

**Published:** 2024-04-22

**Authors:** Shihao Huang, Yingchun Li, Bingjie Wang, Zhihao Zhou, Yonglong Li, Lingjun Shen, Jinge Cong, Liuxin Han, Xudong Xiang, Jiawei Xia, Danhua He, Zhanlin Zhao, Ying Zhou, Qiwen Li, Guanqi Dai, Hanzhang Shen, Taoyan Lin, Aibing Wu, Junshuang Jia, Dong Xiao, Jing Li, Wentao Zhao, Xiaolin Lin

**Affiliations:** 1 Cancer Research Institute, Experimental Education and Administration Center, School of Basic Medical Sciences, Southern Medical University, Guangzhou 510515, China; 2 Southern Medical University Hospital of Integrated Traditional Chinese and Western Medicine, Southern Medical University, Guangzhou 510315, China; 3 Laboratory Animal Management Center, Southern Medical University, Guangzhou 510515, China; 4 Department of Tuberculosis, Yunnan Clinical Medical Center for Infectious Diseases, The Third People's Hospital of Kunming (The Sixth Affiliated Hospital of Dali University), Kunming 650041, China; 5 Yunnan Clinical Medical Center for Infectious Diseases, The Third People’s Hospital of Kunming (The Sixth Affiliated Hospital of Dali University), Kunming 650041, China; 6 Department of Thoracic Surgery, Peking University Cancer Hospital Yunnan (Yunnan Cancer Hospital, The Third Affiliated Hospital of Kunming Medical University), Kunming 650118, China; 7 Department of Gastrointestinal Oncology, Peking University Cancer Hospital Yunnan (Yunnan Cancer Hospital, The Third Affiliated Hospital of Kunming Medical University), Kunming 650118, China; 8 Department of Pharmacy, Nanfang Hospital, Southern Medical University, Guangzhou 510515, China; 9 Central People’s Hospital of Zhanjiang, Zhanjiang 524000, China; 10 Radiotherapy Center, the First People’s Hospital of Chenzhou, Xiangnan University, Chenzhou 423000, China

**Keywords:** METTL3, Alb-iCre mice (GPT), Alb-Cre mice (JAX), acute liver failure (ALF), postnatal lethality

## Abstract

Aim: In 2019, to examine the functions of METTL3 in liver and underlying mechanisms, we generated mice with hepatocyte-specific METTL3 homozygous knockout (METTL3Δhep) by simultaneously crossing METTL3fl/fl mice with Alb-iCre mice (GPT) or Alb-Cre mice (JAX), respectively. In this study, we explored the potential reasons why hepatocyte-specific METTL3 homozygous disruption by Alb-iCre mice (GPT), but not by Alb-Cre mice (JAX), resulted in acute liver failure (ALF) and then postnatal lethality.

Main Methods: Mice with hepatocyte-specific METTL3 knockout were generated by simultaneously crossing METTL3fl/fl mice with Alb-iCre mice (GPT; Strain No. T003814) purchased from the GemPharmatech Co., Ltd., (Nanjing, China) or with Alb-Cre mice (JAX; Strain No. 003574) obtained from The Jackson Laboratory, followed by combined-phenotype analysis. The publicly available RNA-sequencing data deposited in the NCBI Gene Expression Omnibus (GEO) database under the accession No.: GSE198512 (postnatal lethality), GSE197800 (postnatal survival) and GSE176113 (postnatal survival) were mined to explore the potential reasons why hepatocyte-specific METTL3 homozygous deletion by Alb-iCre mice (GPT), but not by Alb-Cre mice (JAX), leads to ALF and then postnatal lethality.

Key Findings: Firstly, we observed that hepatocyte-specific METTL3 homozygous deficiency by Alb-iCre mice (GPT) or by Alb-Cre mice (JAX) caused liver injury, abnormal lipid accumulation and apoptosis. Secondly, we are surprised to find that hepatocyte-specific METTL3 homozygous deletion by Alb-iCre mice (GPT), but not by Alb-Cre mice (JAX), led to ALF and then postnatal lethality. Our findings clearly demonstrated that METTL3Δhep mice (GPT), which are about to die, exhibited the severe destruction of liver histological structure, suggesting that METTL3Δhep mice (GPT) nearly lose normal liver function, which subsequently contributes to ALF, followed by postnatal lethality. Finally, we unexpectedly found that as the compensatory growth responses of hepatocytes to liver injury induced by METTL3Δhep (GPT), the proliferation of METTL3Δhep hepatocytes (GPT), unlike METTL3Δhep hepatocytes (JAX), was not evidenced by the significant increase of Ki67-positive hepatocytes, not accompanied by upregulation of cell-cycle-related genes. Moreover, GO analysis revealed that upregulated genes in METTL3Δhep livers (GPT), unlike METTL3Δhep livers (JAX), are not functionally enriched in terms associated with cell cycle, cell division, mitosis, microtubule cytoskeleton organization, spindle organization, chromatin segregation and organization, and nuclear division, consistent with the loss of compensatory proliferation of METTL3Δhep hepatocytes (GPT) observed *in vivo*. Thus, obviously, the loss of the compensatory growth capacity of METTL3Δhep hepatocytes (GPT) in response to liver injury might contribute to, at least partially, ALF and subsequently postnatal lethality of METTL3Δhep mice (GPT).

Significance: These findings from this study and other labs provide strong evidence that these phenotypes (i.e., ALF and postnatal lethality) of METTL3Δhep mice (GPT) might be not the real functions of METTL3, and closely related with Alb-iCre mice (GPT), suggesting that we should remind researchers to use Alb-iCre mice (GPT) with caution to knockout gene in hepatocytes *in vivo*.

## INTRODUCTION

It is well known that the liver is an essential organ in vertebrates with multiple complex functions, such as material metabolism (e.g., lipid, glucose, vitamin, drug and hormone metabolism) and detoxification [1–4].

The liver plays a unique role in controlling whole body lipid homeostasis, including the synthesis of new fatty acids (FA), hepatic uptake of circulating free FA, FA oxidation, the biosynthesis of triglycerides (TG) from glucose and amino acids in liver, and TG secretion [5], etc, and the disruption of one or more of these pathways essential for the aforementioned complex processes might lead to lipid metabolism abnormality, which is often a major contributing factor to metabolic diseases such as nonalcoholic fatty liver disease (NAFLD) [6].

In recent years, accumulated evidence has illustrated that hepatic cholesterol accumulation, as characterized by increased cholesterol synthesis, elevated uptake from circulating lipoproteins and decreased cholesterol excretion, contributes to the pathogenesis of NAFLD [7–9]. Therefore, identifying the key regulators of hepatic lipid metabolism will be critical for developing effective prevention and treatment approaches for NAFLD.

In recent years, RNA modifications have emerged as a new layer of epigenetic modulation, among which N6-methyl-adenosine (m^6^A) is the most prevalent messenger RNA modification in eukaryotes [10–13]. m^6^A modification is dynamically reversible, and m^6^A is deposited by the m^6^A methyltransferase complex (i.e., METTL3/METTL14/WTAP complex) and erased by m^6^A demethylases (e.g., FTO and ALKBH5) [10–13]. m^6^A modification is recognized by m^6^A reader proteins (i.e., YTHDC1/2, YTHDF1/2/3 and IGF2BP1/2/3), and are thus involved in various steps of RNA metabolism, including the stability, translation, nuclear exportation, splicing, and biogenesis and maturation of m^6^A-containing mRNAs [10–13].

With deeply understanding the biochemical processes of m^6^A modification in the past decade, more and more studies have moved forward to examine the functional significance of m^6^A modification in various physiological and pathological processes, including DNA damage repair, meiosis, circadian clock, and cancer development and progression [10–13]. Recently, increasing evidence has shown that m^6^A modification plays important roles [10–16] in liver development [16–18], liver regeneration and homeostasis [19–21], liver glycogenesis [22], and the development and progression of liver diseases, such as liver injury [17, 23, 24], NAFLD [18, 24, 25], hepatic fibrosis [26], and hepatocellular carcinoma (HCC) [27–32].

In 2019, to investigate the functions of METTL3 in liver development, liver homeostasis maintenance and liver diseases, and underlying mechanisms, we generated mice with hepatocyte-specific METTL3 knockout (METTL3^Δhep^) by simultaneously crossing METTL3^fl/fl^ mice (with loxP sites flanking exons 4) with Alb-iCre mice (GPT; Strain No. T003814) purchased from the GemPharmatech Co., Ltd., (Nanjing, China) or with Alb-Cre mice (JAX; Strain No. 003574) obtained from The Jackson Laboratory. We were surprised to find that hepatocyte-specific METTL3 homozygous knockout by Alb-iCre mice (GPT) resulted in liver injury, acute liver failure (ALF) and then postnatal lethality, whereas hepatocyte-specific METTL3 homozygous deletion by Alb-Cre mice (JAX) led to liver damage, but didn’t cause ALF and postnatal lethality. In this study, we want to explore the potential reasons why hepatocyte-specific homozygous ablation of METTL3 by Alb-iCre mice (GPT), but not by Alb-Cre mice (JAX), caused ALF and then postnatal lethality. Furthermore, based on these findings from this study and other labs, and our in-depth discussion, we remind researchers to use Alb-iCre mice (GPT) with caution to knockout genes in hepatocytes *in vivo*.

## RESULTS

### Generation of hepatocyte-specific METTL3 homozygous knockout (METTL3^Δhep^) mice by Alb-iCre mice (GPT)

**Figure 1:**
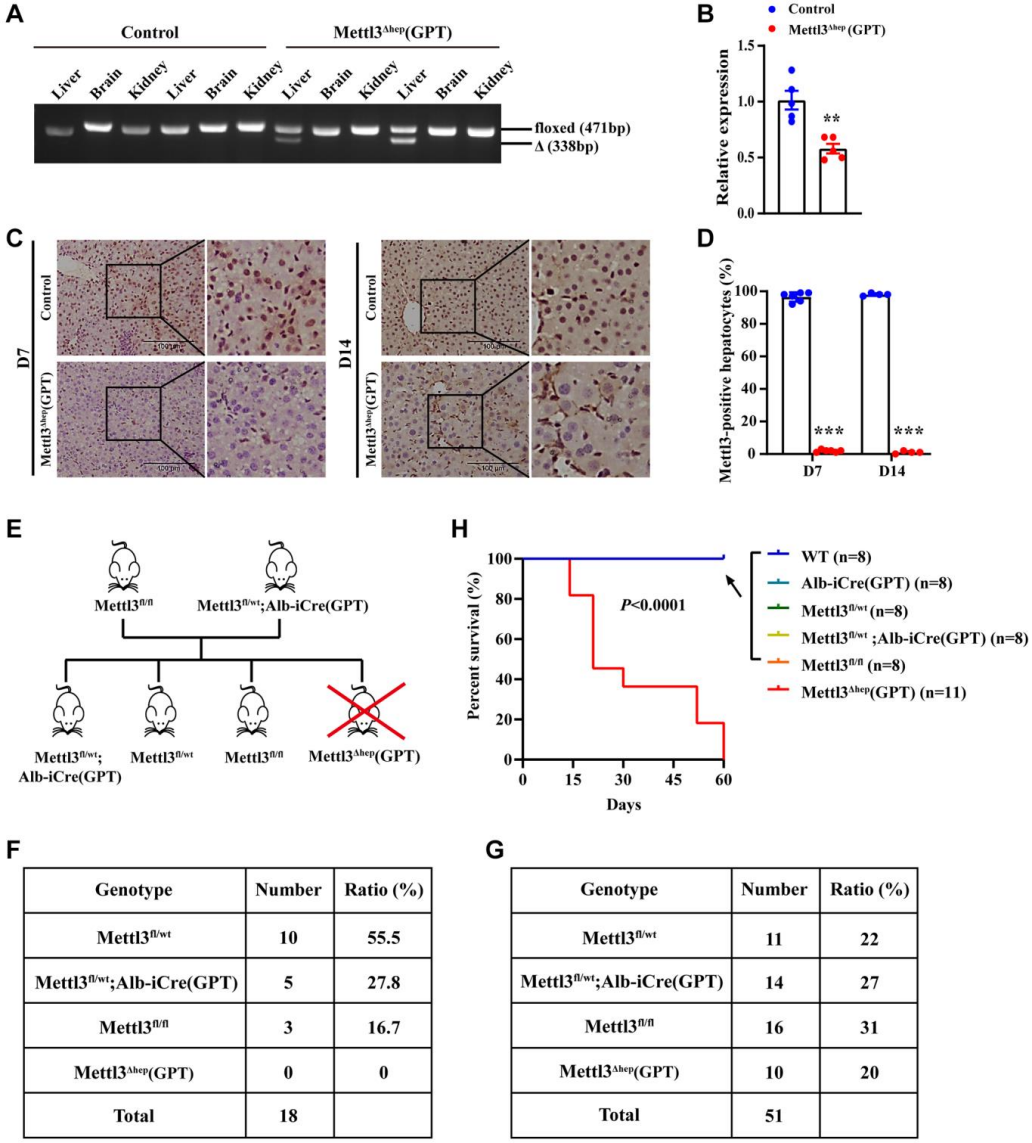
**Homozygous ablation of METTL3 in murine hepatocytes by Alb-iCre mice (GPT) leads to postnatal lethality.** (**A**) Hepatocyte-specific METTL3 homozygous knockout assessed by PCR-based genotyping on genomic DNA collected from the indicated organs of control mice and METTL3^fl/fl^; Alb-iCre mice (GPT) (Referred to as METTL3^Δhep^ mice (GPT)). (**B**) qRT-PCR assay of METTL3 mRNA expression in the livers of control mice and METTL3^Δhep^ mice (GPT). (**C**, **D**) Immunohistochemistry (IHC) staining of METTL3 in the livers of 7- or 14-day-old control mice and METTL3^Δhep^ mice (GPT). The percentages of METTL3-positive hepatocytes were calculated by determining the total number of METTL3-positive hepatocytes divided by the total number of hepatocytes. (**E**) A schematic representation of the offspring with indicated genotypes from intercrossing METTL3^fl/fl^ mice and METTL3^fl/wt^; Alb-iCre (GPT) mice. (**F**) PCR-based genotyping during the late postnatal period displays the absence of offspring with the genotype (i.e., METTL3^Δhep^ (GPT)) from intercrossing METTL3^fl/fl^ mice and METTL3^fl/wt^; Alb-iCre (GPT) mice. (**G**) PCR-based genotyping during the early postnatal period exhibits the number of offspring with indicated genotypes from intercrossing METTL3^fl/fl^ mice and METTL3^fl/wt^; Alb-iCre (GPT) mice. (**H**) Survival curves of WT, Alb-iCre (GPT), METTL3^fl/wt^, METTL3^fl/wt^; Alb-iCre (GPT), METTL3^fl/fl^ and METTL3^Δhep^ (GPT) mice (*n* = 8–11 for each group).

To examine the roles of METTL3 in the physiological and pathological processes of liver and underlying mechanisms, we generated mice with hepatocyte-specific METTL3 homozygous deletion (METTL3^Δhep^) by crossing METTL3^fl/fl^ mice (with loxP sites flanking exons 4) with Alb-iCre mice (GPT; Strain No. T003814) purchased from the GemPharmatech Co., Ltd., (Nanjing, China) (Supplementary Figure 1). The hepatocyte-specific METTL3 homozygous disruption was validated by PCR-based genotyping (Figure 1A). As expected, qRT-PCR and immunochemistry revealed that livers from METTL3^fl/fl^; Alb-iCre mice (GPT) (Referred to as METTL3^Δhep^ mice (GPT)) indicated a significant decrease in mRNA (Figure 1B) and protein (Figure 1C, 1D) levels of METTL3 expression, compared to control mice. Collectively, these findings demonstrate that we successfully generate mice with hepatocyte-specific METTL3 homozygous deficiency by Alb-Cre mice (GPT).

### Hepatocyte-specific METTL3 homozygous disruption by Alb-iCre mice (GPT) causes postnatal lethality

To investigate the functions of METTL3 in liver and the underlying mechanisms, we want to attain the offspring with the genotype (i.e., METTL3^Δhep^ (GPT)) from intercrossing METTL3^fl/fl^ mice and METTL3^fl/wt^; Alb-iCre (GPT) mice. Surprisingly, mice with the genotype (i.e., METTL3^Δhep^ (GPT)) are always absent from the offspring (total: 18 mice) from intercrossing METTL3^fl/fl^ mice and METTL3^fl/wt^; Alb-iCre (GPT) mice while we carried out PCR-based genotyping during the late postnatal period (~3 weeks after birth) (Figure 1E, 1F). Based on this situation, we performed PCR-based genotyping within one week after birth, and we identified mice with the genotype (i.e., METTL3^Δhep^ (GPT)) from the 51 newborn progeny derived from mating METTL3^fl/fl^ mice with METTL3^fl/wt^; Alb-iCre mice (GPT) (Figure 1G). METTL3^Δhep^ mice (GPT) were born at almost expected Mendelian ratios (Figure 1G), excluding the possibility of the prenatal lethality. Furthermore, all the METTL3^Δhep^ mice (GPT) died within 9 weeks after birth, regardless of whether they were male or female, however, Alb-iCre mice (GPT), METTL3^fl/wt^ mice, METTL3^fl/wt^; Alb-iCre (GPT) mice and METTL3^fl/fl^ mice were fertile and survived without discernible defects in development (Figure 1H), suggesting that one allele of METTL3 is enough to maintain the normal development and functions of liver in mice. Together, hepatocyte-specific METTL3 homozygous deficiency in mice by Alb-iCre mice (GPT) results in postnatal lethality within 9 weeks after birth.

### Hepatocyte-specific and homozygous ablation of METTL14 by Alb-iCre mice (GPT) also results in postnatal lethality

To examine the functions of METTL14 in liver and the underlying mechanisms, we want to achieve the offspring with the genotype (i.e., METTL14^fl/fl^; Alb-iCre (GPT): referred to as METTL14^Δhep^ (GPT)) from intercrossing METTL14^fl/fl^ mice and METTL14^fl/wt^; Alb-iCre (GPT) mice (Supplementary Figure 2A). Unexpectedly, mice with the genotype (i.e., METTL14^Δhep^ (GPT)) are always absent from the offspring (total: 39 mice) from intercrossing METTL14^fl/fl^ mice and METTL14^fl/wt^; Alb-iCre (GPT) mice while we carried out PCR-based genotyping during the late postnatal period (~ 3 weeks after birth) (Supplementary Figure 2B). Based on this situation, we performed PCR-based genotyping within one week after birth, and we identified mice with the genotype (i.e., METTL14^Δhep^ (GPT)) from the 21 newborn progeny derived from mating METTL14^fl/fl^ mice with METTL14^fl/wt^; Alb-iCre (GPT) mice (Supplementary Figure 2C). METTL14^Δhep^ mice (GPT) were born at almost expected Mendelian frequencies (Supplementary Figure 2C), excluding the possibility of the prenatal lethality. Moreover, METTL14^fl/wt^ mice, METTL14^fl/fl^ mice and METTL14^fl/wt^; Alb-iCre (GPT) mice were fertile and survived without discernible defects in development (Supplementary Figure 2). Together, hepatocyte-specific METTL14 homozygous deficiency in mice by Alb-iCre mice (GPT) also causes postnatal lethality.

### Hepatocyte-specific METTL3 homozygous deficiency by Alb-iCre mice (GPT) leads to liver injury, apoptosis and acute liver failure (ALF)

**Figure 2:**
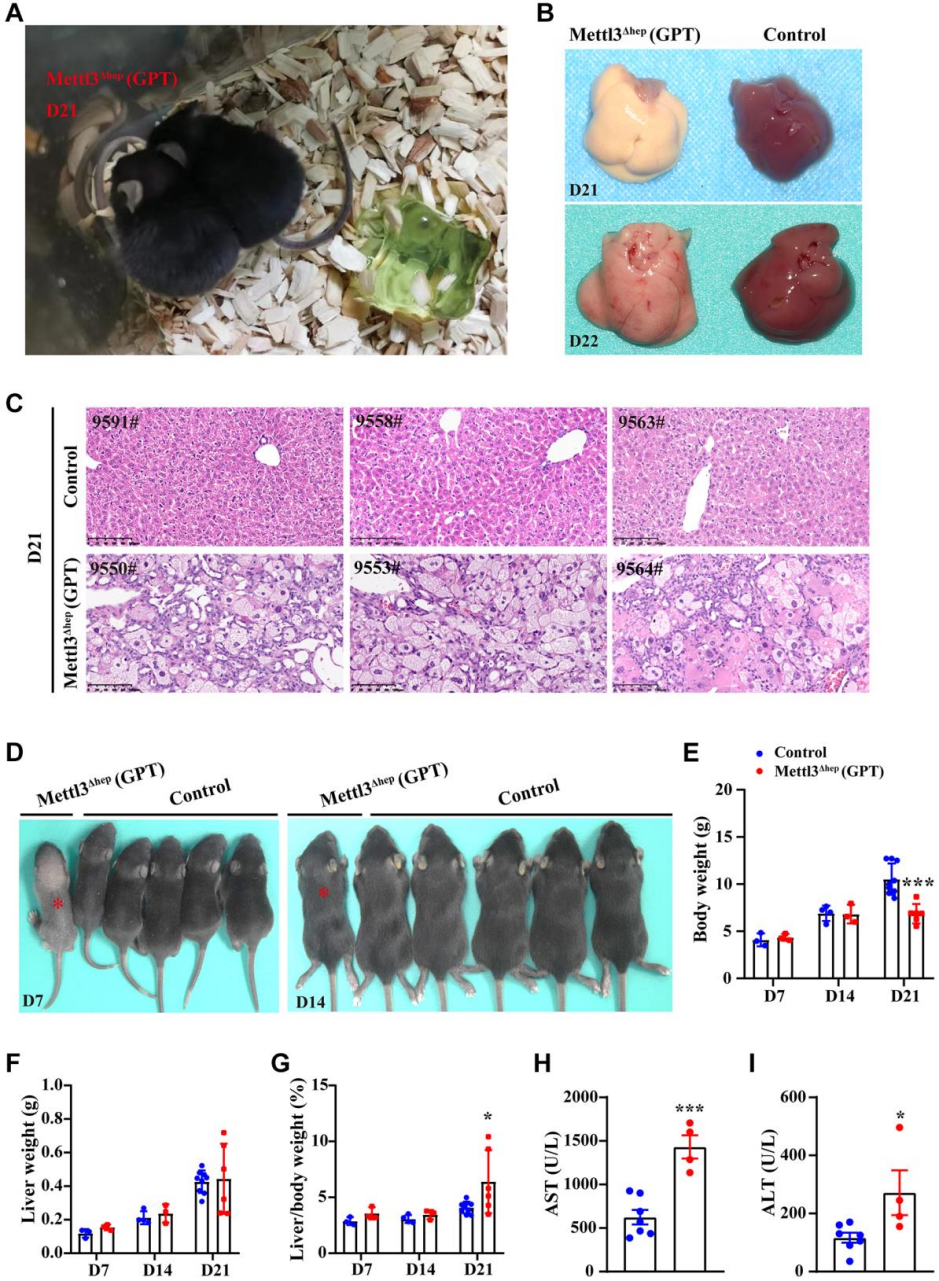
**Homozygous deletion of METTL3 in murine hepatocytes by Alb-iCre mice (GPT) results in liver injury and acute liver failure (ALF).** (**A**) Representative appearance of METTL3^Δhep^ mice (GPT) at 21 days after birth. (**B**) Representative gross appearance of livers from control mice and METTL3^Δhep^ mice (GPT) at 21 or 22 days postnatally. (**C**) Representative H&E staining photographs of liver sections from control mice and METTL3^Δhep^ mice (GPT) at 21 days postnatally. Scale bar = 100 μm. (**D**) Representative appearance of control mice and METTL3^Δhep^ mice (GPT) at 7 or 14 days after birth. (**E**–**G**) Body weight (**E**), liver weight (**F**) and liver-to-body weight ratio (**G**) of control mice and METTL3^Δhep^ mice (GPT) at 7, 14 or 21 days after birth. (**H**, **I**) Serum levels of AST (**H**) and ALT (**I**) of control mice and METTL3^Δhep^ mice (GPT).

Subsequently, we aim to determine the possible reasons for postnatal lethality induced by hepatocyte-specific METTL3 homozygous deletion by Alb-iCre mice (GPT). Firstly, we dissected the livers of control mice and METTL3^Δhep^ mice (GPT) before death. Before METTL3^Δhep^ mice (GPT) were about to die, we found that all the METTL3^Δhep^ mice (GPT) grow thin and visibly waste away, with a matte coat, and presented slightly swollen abdomen, decreased body temperature, slowly the energetic dispirited, the slow reaction, lack of movement, hugs together, and decreased appetite (Figure 2A). By careful dissection of the mice at 21 or 22 days after birth, we found that the livers of METTL3^Δhep^ mice (GPT) were relatively stiff, and they appeared a pale color (Figure 2B). The destruction of normal liver histological structure of METTL3^Δhep^ mice (GPT) was quite evident upon microscopic analysis (Figure 2C). The hepatic lobule of control mice showed the clear and normal structure of hepatic cords and hepatic sinusoids, with hepatic sinusoids and cords arranging radially around the central vein, and the liver of control mice exhibited the clear structure of arteriovenous and biliary ducts in the portal area (Figure 2C). Conversely, compared with control mice, the liver histological changes of METTL3^Δhep^ mice (GPT) included severe hepatocyte edema, and eosinophilic necrosis (i.e., apoptosis) in scattered hepatocytes (Figure 2C). Moreover, the architecture of hepatic cords and hepatic sinusoids of METTL3^Δhep^ mice (GPT) disappeared due to the compression from severe hepatocyte edema and the liver of METTL3^Δhep^ mice (GPT) displayed the disordered structure in the portal area (Figure 2C). METTL3^Δhep^ mice (GPT) presented the hyperplasia of bile ducts in liver, with partial hyperplasia of bile ducts spreading towards the center of the lobules (Figure 2C). Together, our findings clearly demonstrate that METTL3^Δhep^ mice (GPT), which are about to die, exhibit the severe destruction of liver histological structure, suggesting that METTL3^Δhep^ mice (GPT) nearly lose normal liver function.

To fully study the exact role of METTL3 in liver organogenesis, we further dissected the livers of control mice and METTL3^Δhep^ mice (GPT) at different time points after birth. The body weight (Figure 2D, 2E), liver weight (Figure 2F) and liver-to-body weight ratio (Figure 2G) were comparable between control mice and METTL3^Δhep^ mice (GPT) at both 7 and 14 days postnatally. METTL3^Δhep^ mice (GPT) at 21 days postnatally are lighter in body weight than control mice (Figure 2E), while the liver weight to body weight ratio of METTL3^Δhep^ mice (GPT) is slightly increased (Figure 2G). Moreover, compared with control group, the activities of serum alanine aminotransferase (ALT) and aspartate aminotransferase (AST) in METTL3^Δhep^ mice (GPT) were significantly increased (Figure 2H, 2I), indicating that hepatocyte-deletion of METTL3 by Alb-iCre mice (GPT) results in the progressive liver injury.

**Figure 3:**
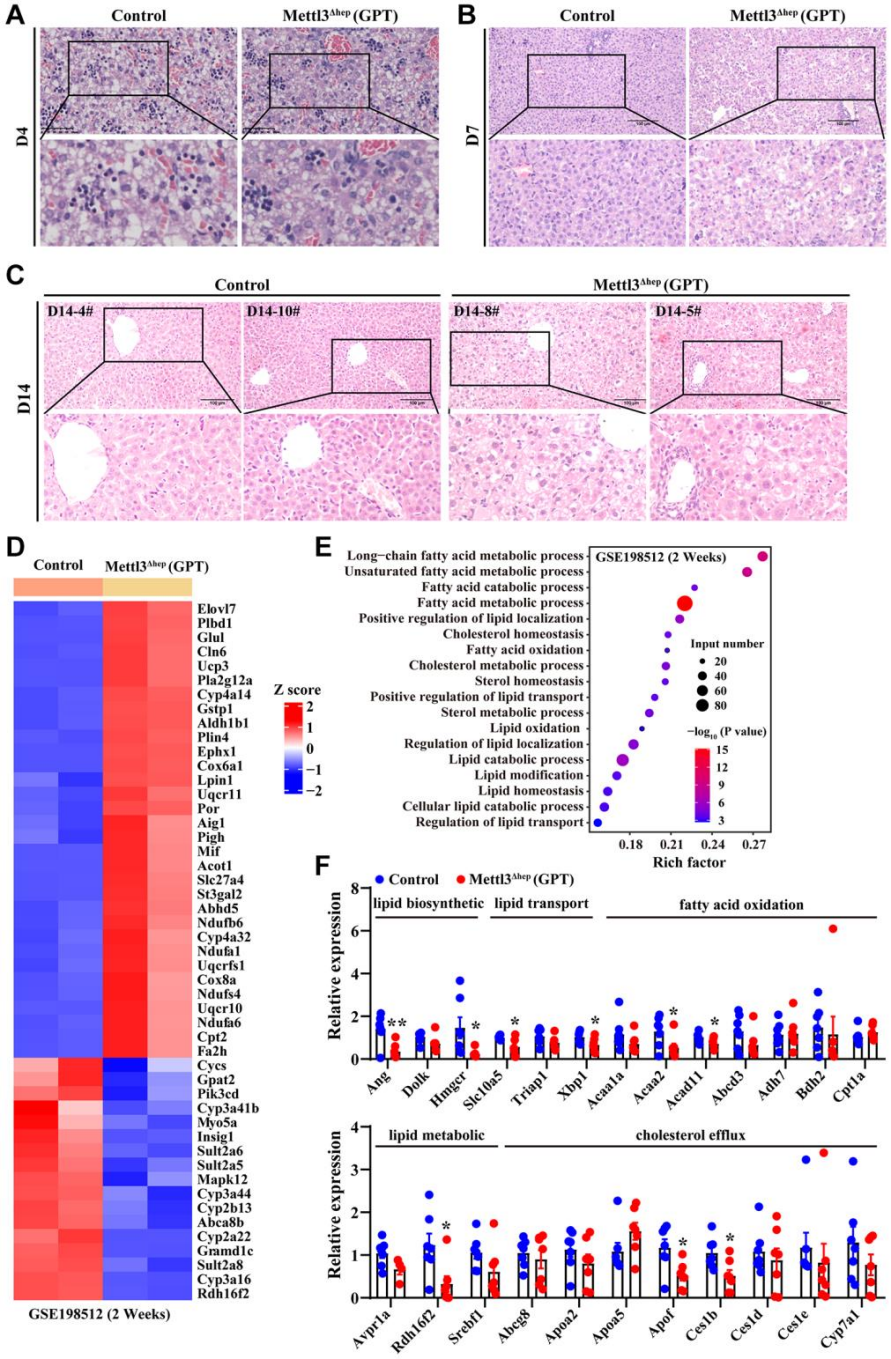
**Hepatic METTL3 homozygous knockout by Alb-iCre mice (GPT) induces abnormal lipid accumulation in mouse hepatocytes.** (**A**–**C**) Representative H&E staining photographs of liver sections from control mice and METTL3^Δhep^ mice (GPT) at 4 (**A**), 7 (**B**) and 14 (**C**) days postnatally. Scale bar = 100 μm. (**D**) Heatmap depicts the differential expression of hepatic lipid metabolism-related genes from RNA-seq results deposited in NCBI GEO under the accession number GSE198512 [17]. In the cluster heatmap, class comparison and hierarchical clustering of differentially expressed genes (DEGs) involved in hepatic lipid metabolism were performed between control mice and METTL3^Δhep^ mice (GPT) at 2 weeks after birth. Genes with increased and reduced expressions are shown in red and blue, respectively. (**E**) GO analysis of up- and downregulated genes related with hepatic lipid metabolism (from RNA-seq data deposited in GEO under accession number GSE198512) in the liver of control mice and METTL3^Δhep^ mice (GPT) at 2 weeks after birth. (**F**) qRT-PCR analysis of the mRNA expression of genes related with to fatty acid oxidation, cholesterol efflux, lipid metabolic process, lipid transport and lipid biosynthetic process in the livers of METTL3^Δhep^ mice (GPT).

To fully delineate the exact functions of METTL3 in liver organogenesis, we further dissected the livers of control mice and METTL3^Δhep^ mice (GPT) at different time points postnatally. The destruction of liver histological structure of METTL3^Δhep^ mice (GPT) at 4, 7 and 14 days after birth was quite evident upon microscopic analysis (Figure 3A–3C). H&E staining of liver tissues of control mice at 4, 7 and 14 days postnatally displayed the clear and normal structure of hepatic cords and hepatic sinusoids, and normal hepatocyte morphology (Figure 3A–3C). Conversely, compared with control mice, we found that the hepatic lobule of 4-day-old METTL3^Δhep^ mice (GPT) showed mild-to-moderate hepatocyte edema and significantly compressed hepatic sinusoids (Figure 3A). In the liver of 7-day-old METTL3^Δhep^ mice (GPT), the fat vacuoles of varying sizes were observed in hepatocytes, and hepatic cords and hepatic sinusoids in liver region with more severe steatosis disappeared due to the compression from severe hepatocytes, while liver region with lighter steatosis exhibited the normal architecture of hepatic cords and hepatic sinusoids (Figure 3B). In the liver of 14-day-old METTL3^Δhep^ mice (GPT), microscopic examination revealed hypertrophy of hepatocytes in METTL3^Δhep^ livers (GPT), with both cell and nuclear size enlarged (Figure 3C). Mild edema and fat vacuoles were observed in the cytoplasm of METTL3^Δhep^ hepatocytes (GPT), and the structure of hepatic cords and hepatic sinusoids of METTL3^Δhep^ mice (GPT) disappeared due to the compression from hepatocyte edema (Figure 3C). Additionally, there is no difference in extramedullary hematopoiesis diffusely distributed in the liver between control mice and METTL3^Δhep^ mice (GPT) at 4 and 7 days postnatally (Figure 3A, 3B), while at 14 days after birth, no extramedullary hematopoiesis was observed in the liver of both control mice and METTL3^Δhep^ mice (GPT) (Figure 3C).

**Figure 4:**
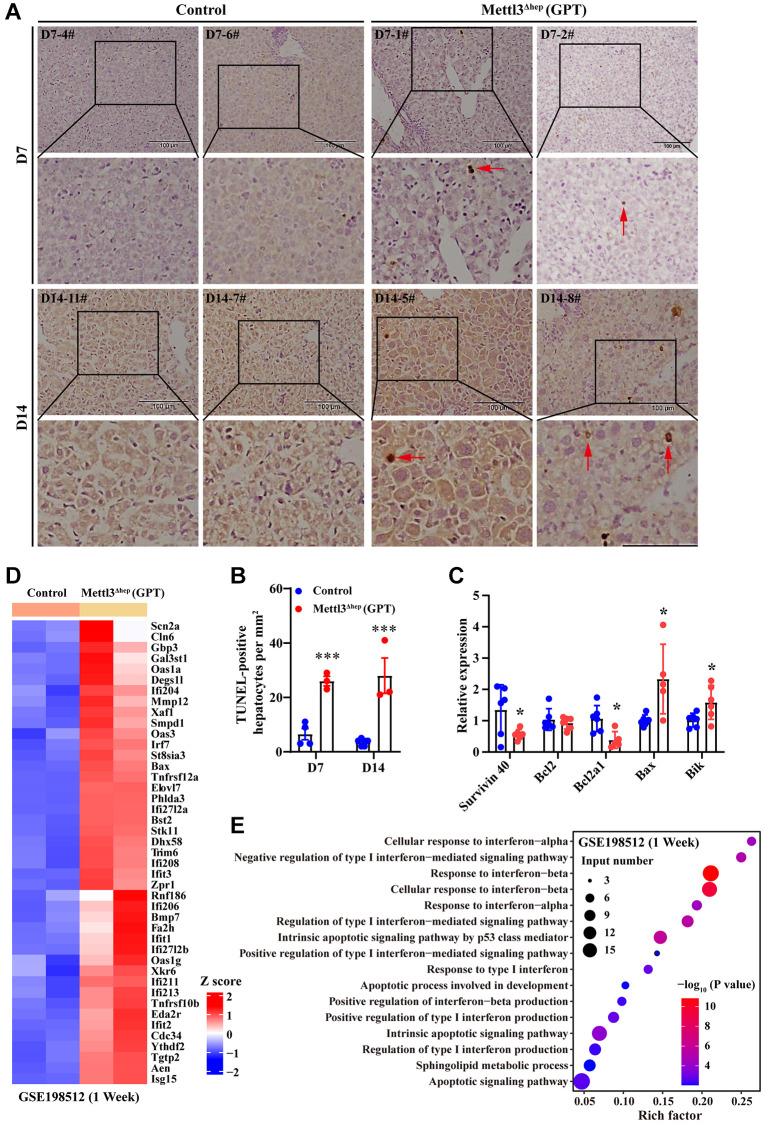
**Hepatocyte-specific METTL3 homozygous ablation by Alb-iCre mice (GPT) causes apoptosis in mouse hepatocytes.** (**A**, **B**) Representative TUNEL staining photographs (**A**) and quantification of TUNEL-positive hepatocytes of paraffin-embedded liver sections from control mice and METTL3^Δhep^ mice (GPT) at 7 or 14 days after birth. Scale bar = 100 μm. (**C**) qRT-PCR analysis of the expression of apoptosis-related genes in the liver of METTL3^Δhep^ mice (GPT). (**D**) Hierarchical clustering of differentially expressed genes (DEG) related to apoptosis in the liver of control mice and METTL3^Δhep^ mice (GPT) at 1 week after birth from RNA-seq data deposited in GEO under accession number GSE198512 [17]. In the cluster heatmap, class comparison and hierarchical clustering of DEGs involved in apoptosis in mouse livers were performed between control mice and METTL3^Δhep^ mice (GPT) at 7 days after birth. Genes with increased and reduced expressions are shown in red and blue, respectively. (**E**) GO analysis of up- and downregulated genes related to apoptosis (from RNA-seq data deposited in GEO under accession number GSE198512) in the liver of control mice and METTL3^Δhep^ mice (GPT) at 1 week after birth.

As described above, the eosinophilic necrosis (i.e., apoptosis) was observed in scattered hepatocytes of METTL3^Δhep^ mice (GPT) (Figure 2C and Figure 3A–3C). Therefore, we evaluated whether these hepatocytes of METTL3^Δhep^ mice (GPT) underwent apoptosis induced by hepatocyte-specific METTL3 loss. The nuclei of >0.5%–2.0% of the hepatocytes of METTL3^Δhep^ mice (GPT) at 7 or 14 days after birth were condensed because of apoptosis, which was demonstrated by TUNEL staining (Figure 4A, 4B). Furthermore, the qRT-PCR results revealed the downregulation of anti-apoptosis genes such as survivin 40 and Bcl2a1, and the upregulation of pro-apoptosis genes such as Bax and Bik (Figure 4C).

RNA-seq results from METTL3^Δhep^ mouse liver (GPT) revealed the significant upregulation of a number of pro-apoptosis and interferon response genes such as Bax, Tnfrsf10b, Tnfrsf12a, Smpd1, Ifi204, Ifi206, Ifi208, Ifi211, Ifi213, Ifi27l2a, Ifi27l2b, Ifit1, Ifit3, Irf7, Isg15, Oas1a, Oas1g and Oas3 (Figure 4D and Supplementary Table 1). All GO terms representing biological processes listed in Figure 4E and Supplementary Table 2 were related to apoptosis, including apoptotic signaling pathway, intrinsic apoptotic signaling pathway, intrinsic apoptotic signaling pathway by p53 class mediator, apoptotic process involved in development and sphingolipid metabolic process, and interferon response, including response to interferon-alpha, response to interferon-beta, response to type I interferon, cellular response to interferon-alpha, cellular response to interferon-beta, regulation of type I interferon-mediated signaling pathway, positive regulation of type I interferon-mediated signaling pathway, negative regulation of type I interferon-mediated signaling pathway, regulation of type I interferon production, positive regulation of type I interferon production and positive regulation of interferon-beta production.

Based on our above-mentioned findings, we believe that ALF might be the major cause of mortality among METTL3^Δhep^ mice (GPT).

### Hepatic METTL3 homozygous deletion by Alb-iCre mice (GPT) causes abnormal lipid accumulation in mouse hepatocytes

We found that the liver of 1-week-old METTL3^Δhep^ mice (GPT) displayed a more yellowish appearance than that in the control group. This difference became more pronounced at 2 weeks postnatally. Consistent with this, H&E staining of liver tissues of METTL3^Δhep^ mice (GPT) at 1 week and 2 weeks after birth showed that the fat vacuoles of varying sizes were observed in METTL3^Δhep^ hepatocytes with diffuse and severe steatosis (Figure 3B, 3C). Collectively, these findings suggest abnormal lipid accumulation in the hepatocytes of 7-day-old and 14-day-old METTL3^Δhep^ mice (GPT).

To dissect the molecular events underlying the abnormal lipid accumulation in the hepatocytes of 14-day-old METTL3^Δhep^ mice (GPT), we further analyzed a publicly available gene expression dataset (from GEO database: GSE198512) of the liver of 14-day-old METTL3^Δhep^ mice (GPT). As we expected, GO analysis of the 2312 genes displaying significant changes in the expression of 14-day-old METTL3^Δhep^ mouse liver (GPT) demonstrated a dramatic enrichment for 49 genes (up-regulated: 32; down-regulated: 17) (Figure 3D and Supplementary Table 3) with functions typically associated with the abnormalities in hepatic lipid metabolism including long-chain fatty acid metabolic process, unsaturated fatty acid metabolic process, fatty acid catabolic process, fatty acid metabolic process, positive regulation of lipid localization, cholesterol homeostasis, fatty acid oxidation, cholesterol metabolic process, sterol homeostasis, positive regulation of lipid transport, sterol metabolic process, lipid oxidation, regulation of lipid localization, lipid catabolic process, lipid modification, lipid homeostasis, cellular lipid catabolic process and regulation of lipid transport (Figure 3E and Supplementary Table 4).

Additionally, to help further elucidate the mechanisms underlying the abnormal lipid accumulation in the hepatocytes of METTL3^Δhep^ mice (GPT), qRT-PCR assay was performed to further analyze the expressions of hepatic lipid metabolism genes. As expected, most of the genes related with fatty acid oxidation (i.e., Acaa2 and Acad11), cholesterol efflux (i.e., Apof and Ces1b), lipid metabolic process (i.e., Rdh16f2), lipid transport (i.e., Slc10a5 and Xbp1), and lipid biosynthetic process (i.e., Ang and Hmgcr) were significantly altered in the livers of METTL3^Δhep^ mice (GPT) (Figure 3F). Collectively, RNA-seq and qRT-PCR data reveal the corresponding altered expression profile of hepatic genes with functions typically associated with hepatic lipid metabolism, which is likely at least partially responsible for the abnormal lipid accumulation observed in the hepatocytes of METTL3^Δhep^ mice (GPT).

### Generation of hepatocyte-specific METTL3 homozygous knockout (METTL3^Δhep^) mice by Alb-Cre mice (JAX)

**Figure 5:**
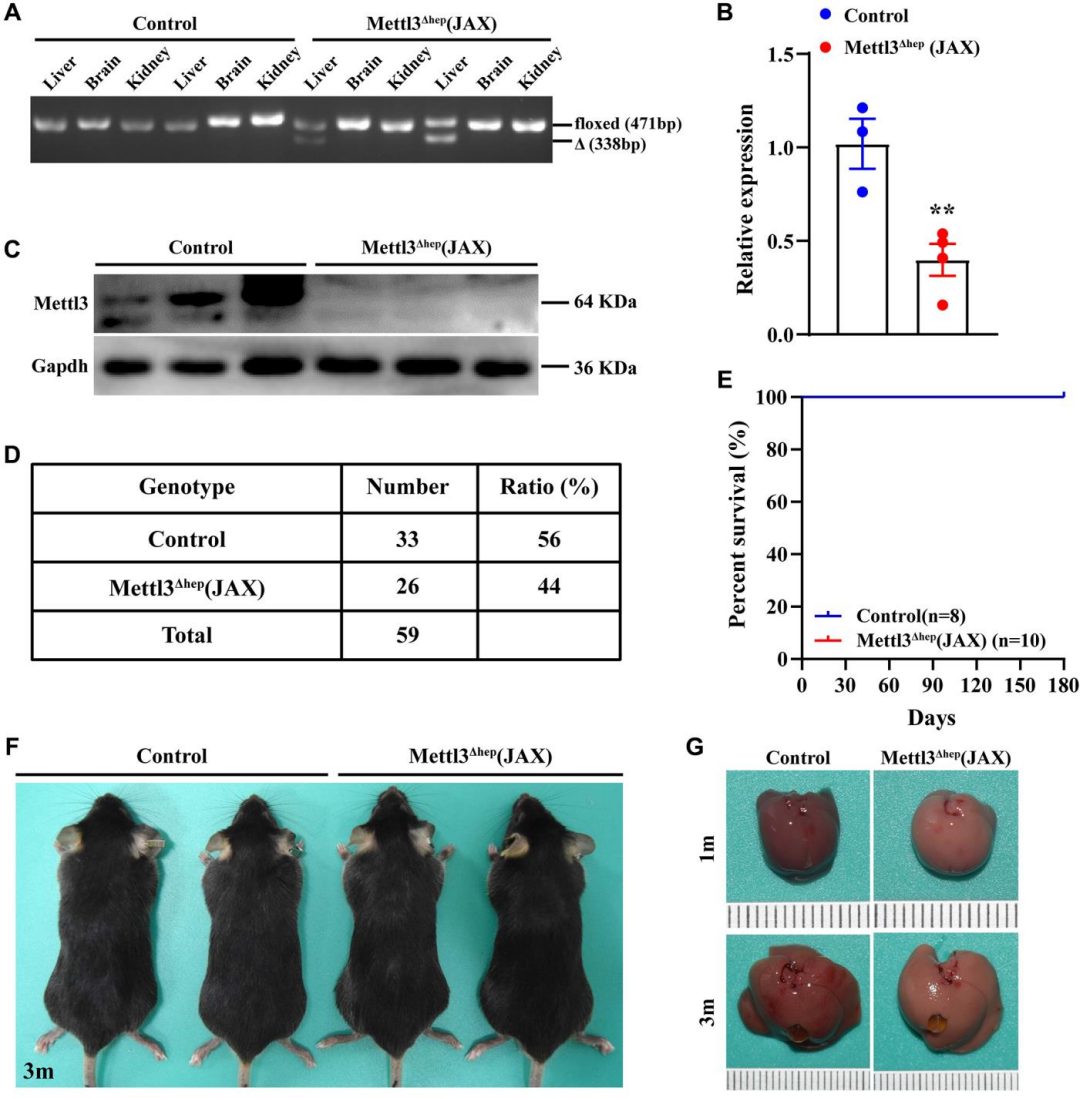
**Homozygous deletion of METTL3 in murine hepatocytes by Alb-Cre mice (JAX) does not lead to postnatal lethality.** (**A**) Hepatocyte-specific METTL3 homozygous knockout assessed by PCR-based genotyping on genomic DNA collected from the indicated organs of control mice and METTL3^fl/fl^; Alb-Cre mice (JAX) (referred to as METTL3^Δhep^ (JAX)). (**B**, **C**) qRT-PCR (**B**) and Western blot assay (**C**) of METTL3 expression in the livers of control mice and METTL3^Δhep^ mice (JAX). (**D**) PCR-based genotyping during the late postnatal period exhibits the number of offspring with indicated genotypes from intercrossing METTL3^fl/fl^ mice and METTL3^fl/fl^; Alb-Cre (JAX) mice. (**E**) Survival curves of control mice and METTL3^Δhep^ mice (JAX) (*n* = 8–10 for each group). (**F**) Representative appearance of control mice and METTL3^Δhep^ mice (JAX) at 3 months after birth. (**G**) Representative gross appearance of livers from control mice and METTL3^Δhep^ mice (JAX) at 1 month or 3 months postnatally.

To explore the functions of METTL3 involved in the physiological and pathological processes of liver and underlying mechanisms, we also produced mice with hepatocyte-specific and homozygous knockout of METTL3 (METTL3^Δhep^) by crossing METTL3^fl/fl^ mice (with loxP sites flanking exons 4) with Alb-Cre mice (JAX; Strain No. 003574) obtained from The Jackson Laboratory (Supplementary Figure 3). The hepatocyte-specific METTL3 homozygous deletion was validated by PCR-based genotyping (Figure 5A). As expected, qRT-PCR and Western blot illustrated that livers from METTL3^fl/fl^; Alb-Cre mice (JAX) (Referred to as METTL3^Δhep^ mice (JAX)) showed a dramatic decrease in mRNA (Figure 5B) and protein (Figure 5C) levels of METTL3 expression, compared to control mice. Together, we also generate hepatocyte-specific METTL3 homozygous knockout mice by Alb-Cre mice (JAX).

Furthermore, both control mice (i.e., METTL3^fl/fl^ mice) and METTL3^Δhep^ mice (JAX) survived (Figure 5D, 5E) without discernible defects in development (Figure 5F), while both control mice (i.e., METTL14^fl/fl^ mice) and METTL14^Δhep^ mice (JAX) survived without discernible defects in development (Supplementary Figure 4). Collectively, our results demonstrate that hepatocyte-specific and homozygous ablation of METTL3 or METTL14 in mice by Alb-Cre mice (JAX) does not lead to postnatal lethality.

### Hepatocyte-specific METTL3 homozygous deficiency by Alb-Cre mice (JAX) causes liver injury and apoptosis

To determine the functions of METTL3 in the liver, we dissected the livers of control mice and METTL3^Δhep^ mice (JAX) at 1 month and 3 months after birth. Our previous study revealed that the body weight, liver weight and liver-to-body weight ratio are comparable between control mice and METTL3^Δhep^ mice (JAX) at both 1 month and 3 months after birth [33]. In addition, compared with control mice, the activities of serum ALT and AST in METTL3^Δhep^ mice (JAX) were notably elevated [33], indicating that hepatocyte-specific METTL3 homozygous ablation by Alb-Cre mice (JAX) leads to the progressive liver damage.

**Figure 6:**
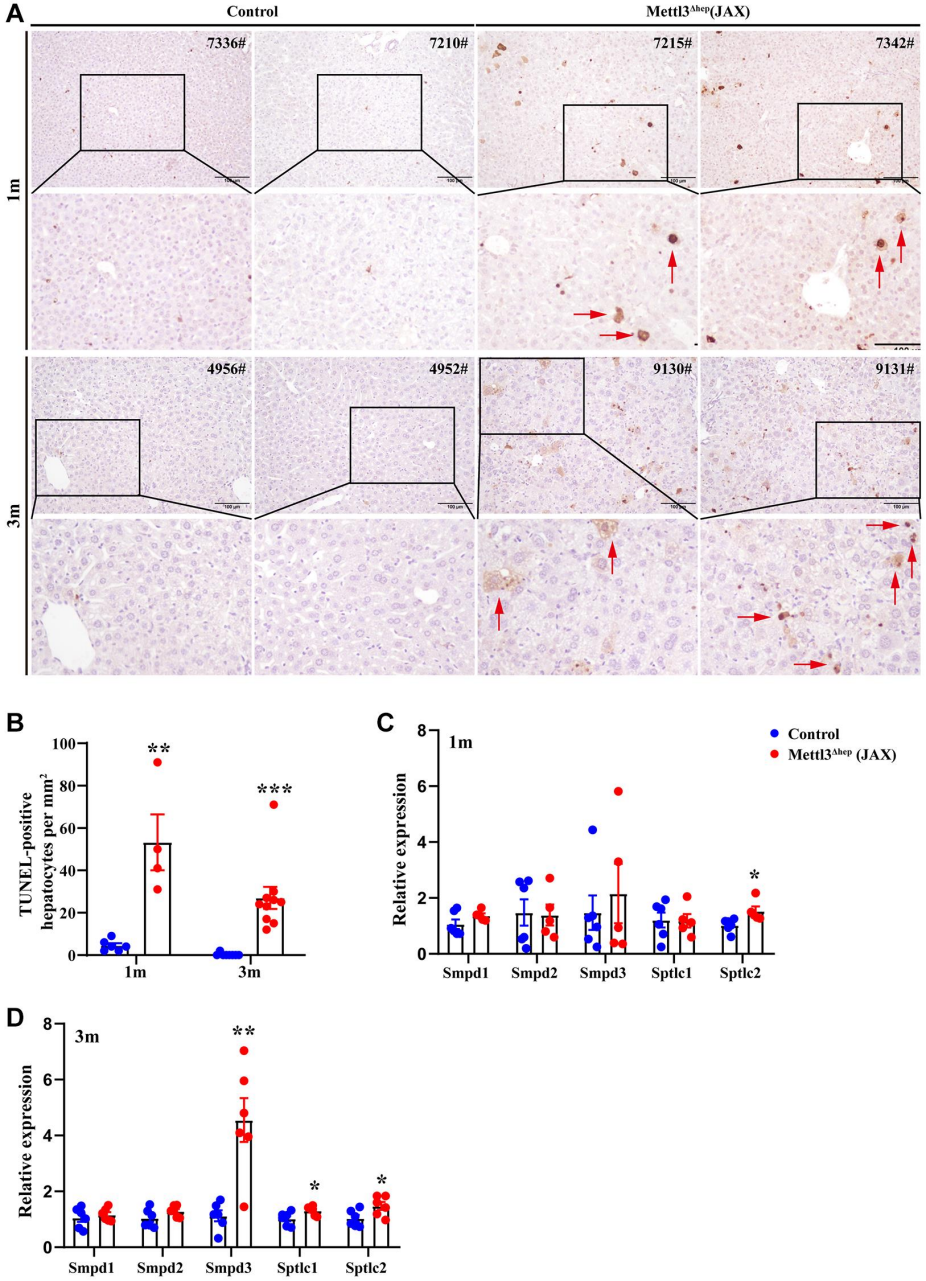
**Hepatocyte-specific METTL3 homozygous ablation by Alb-Cre mice (JAX) results in apoptosis in mouse hepatocytes.** (**A**) Representative TUNEL staining images of paraffin-embedded liver sections from control mice and METTL3^Δhep^ mice (JAX) at 1 month or 3 months after birth. Scale bar = 100 μm. (**B**) Quantification of TUNEL-positive hepatocytes in paraffin-embedded liver sections from control mice and METTL3^Δhep^ mice (JAX). (**C**, **D**) qRT-PCR analysis of the expression of genes related with sphingolipid metabolism in the liver of METTL3^Δhep^ mice (JAX).

As described above, the eosinophilic necrosis (i.e., apoptosis) was observed in scattered hepatocytes of METTL3^Δhep^ mice (JAX) (Figure 6A). Therefore, we evaluated whether these hepatocytes of METTL3^Δhep^ mice (JAX) underwent apoptosis induced by hepatocyte-specific METTL3 loss. The nuclei of >0.6–4% of the hepatocytes of METTL3^Δhep^ mice (JAX) at 1 month or 3 months after birth were condensed because of apoptosis, which was demonstrated by TUNEL staining (Figure 6A, 6B).

Given that perturbation of sphingolipid metabolism can elicit tissue damage characterized by apoptotic cell death [34–36], we then focused on genes related with sphingolipid metabolism and verified the dramatical upregulation of transcripts implicated in sphingolipid metabolism in METTL3^Δhep^ livers (JAX) by qRT–PCR (Figure 6C, 6D). Notably, mRNA levels of Smpd3, which catalyse ceramide generation by hydrolysis of sphingomyelin (SM), and Sptlc1/2, encoding the rate-limiting enzymes of de novo ceramide synthesis, were strongly upregulated in METTL3^Δhep^ livers (JAX) (Figure 6C, 6D). Among these, Smpd3, the neutral sphingomyelinase, was the most strongly upregulated (Figure 6D).

### Hepatocyte-specific METTL3 homozygous knockout by Alb-Cre mice (JAX) induces hepatic lipid metabolism disorder

**Figure 7:**
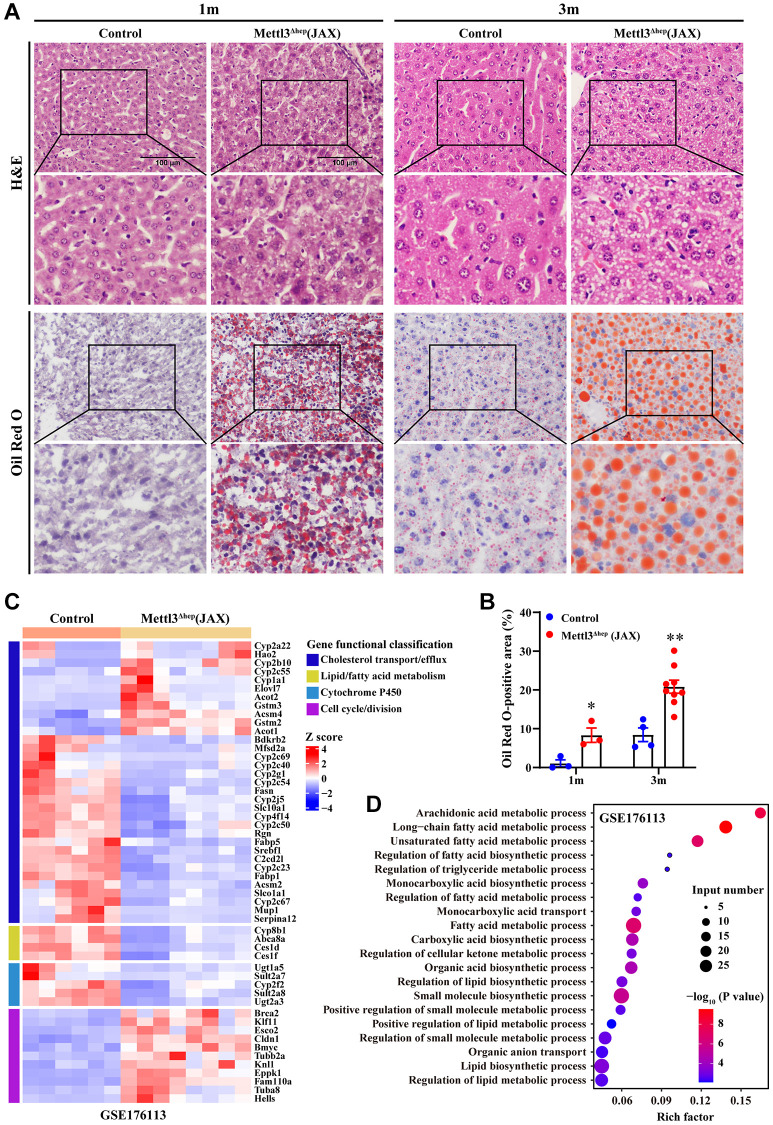
**Hepatic METTL3 homozygous knockout by Alb-Cre mice (JAX) induces lipid accumulation in mouse hepatocytes.** (**A**) Representative images of H&E (upper panel A) and Oil-Red-O (ORO) (lower panel A) staining of liver sections from control mice and METTL3^Δhep^ mice (JAX) at 1 month and 3 months postnatally. Scale bar = 100 μm. (**B**) Quantitative analysis of ORO staining-positive areas of frozen liver sections (shown in Figure 7A, lower panel A) relative to total liver section areas. ORO-positive areas were quantified for each of the five random images using ImageJ Software. (**C**) Heatmap (RNA-seq data deposited in NCBI GEO under the accession number GSE176113 [20]) depicts the differential expression of genes involved in cell cycle, hepatic lipid metabolism and cytochrome P450. In the cluster heatmap, class comparison and hierarchical clustering of differentially expressed genes (DEGs) involved in cell cycle, hepatic lipid metabolism and cytochrome P450 were performed between control mice and METTL3^Δhep^ mice (JAX). Genes with increased and reduced expressions are shown in red and blue, respectively. (**D**) GO analysis of up- and downregulated genes derived from RNA-seq data deposited in NCBI GEO under the accession number GSE176113 [20] related with cell cycle, hepatic lipid metabolism and cytochrome P450 in the liver of control mice and METTL3^Δhep^ mice (JAX).

The livers of 1-month-old and 3-month-old METTL3^Δhep^ mice (JAX) exhibited a more yellowish appearance than in the control group (Figure 5G). Consistent with this, H&E staining of liver tissues of METTL3^Δhep^ mice (JAX) at 1 month and 3 months after birth demonstrated the diffuse microsteatosis of hepatocytes (Figure 7A). Furthermore, liver tissue slices stained with Oil-Red-O (ORO) also revealed that more red lipid droplets were deposited in the hepatocytes of METTL3^Δhep^ mice (JAX) at both timepoints (Figure 7A, 7B). Summarily, these findings suggest abnormal lipid accumulation in the hepatocytes of METTL3^Δhep^ mice (JAX).

To examine the molecular mechanisms underlying abnormal lipid accumulation observed in the hepatocytes of METTL3^Δhep^ mice (JAX), we further analyzed a publicly available gene expression dataset (from GEO database: GSE176113) of the liver of METTL3^Δhep^ mice (JAX). As we expected, GO analysis of the 495 genes displaying significant changes in the expression of METTL3^Δhep^ mouse liver (JAX) demonstrated a dramatic enrichment for 80 genes (up-regulated: 49; down-regulated: 31) (Figure 7C and Supplementary Table 5) with functions typically associated with the abnormalities in hepatic lipid metabolism including long-chain fatty acid metabolic process, unsaturated fatty acid metabolic process, regulation of fatty acid biosynthetic process, regulation of triglyceride metabolic process, regulation of fatty acid metabolic process, fatty acid metabolic process, regulation of lipid biosynthetic process, positive regulation of lipid metabolic process, lipid biosynthetic process and regulation of lipid metabolic process (Figure 7D and Supplementary Table 6). In conclusion, our results suggest that hepatocyte-specific METTL3 homozygous knockout by Alb-Cre mice (JAX) leads to the corresponding altered expression of hepatic lipid metabolism genes, which might account for the abnormal lipid accumulation in the hepatocytes of METTL3^Δhep^ mice (JAX).

### The loss of the compensatory growth responses of METTL3^Δhep^ hepatocytes (GPT) to liver injury contributes to, at least partially, ALF and then postnatal lethality of METTL3^Δhep^ mice (GPT)

**Figure 8:**
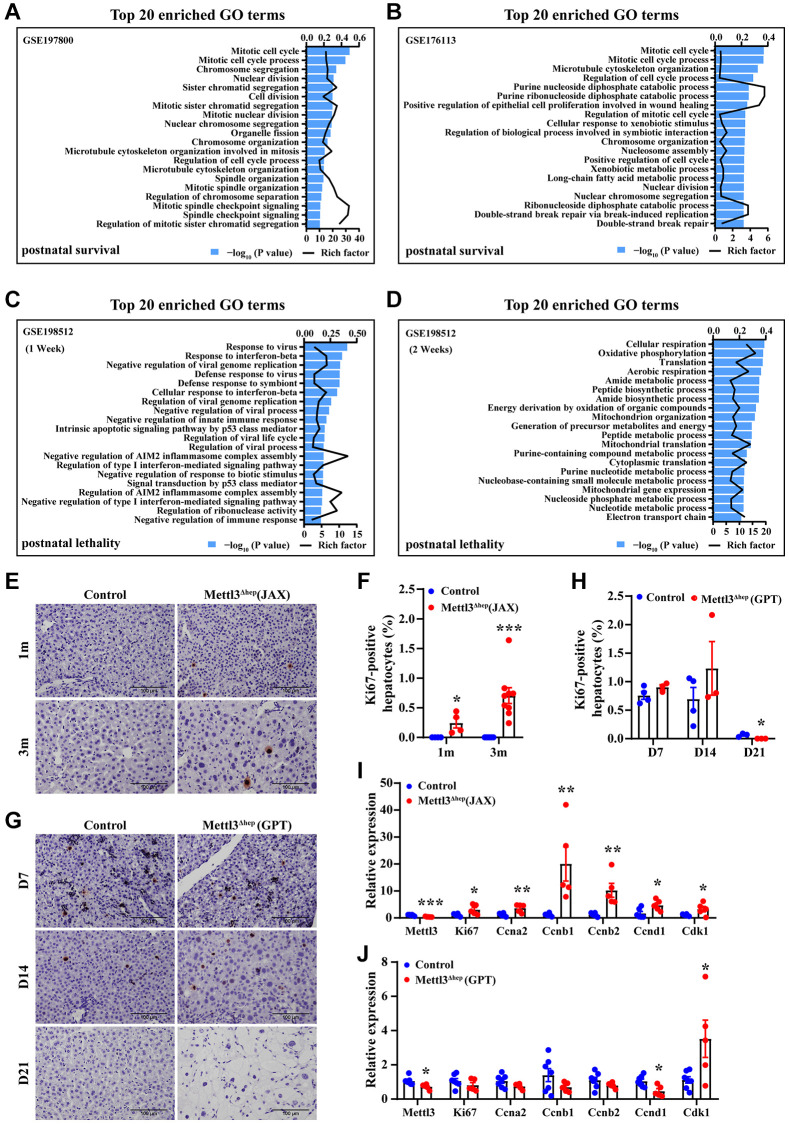
**The loss of the compensatory growth responses of METTL3^Δhep^ hepatocytes (GPT) to liver injury induced by METTL3^Δhep^ (GPT).** (**A**–**D**) The histogram of GO term enrichment analyses based on the GO term of top20 enriched biological process for up-regulated DEGs according to the significance of enrichment (*P*-value). GO analysis of up-regulated genes in the liver of control mice and METTL3^Δhep^ mice (JAX) was performed by using RNA-seq data deposited in NCBI GEO under the accession number GSE197800 [16] (**A**) and GSE176113 [20] (**B**), respectively, while GO analysis of up-regulated genes in the liver of control mice and METTL3^Δhep^ mice (GPT) at 1 week (**C**) and 2 weeks (**D**) after birth was performed by using RNA-seq data deposited in NCBI GEO under the accession number GSE198512 (**C**, **D**). (**E**, **F**) IHC of Ki67 in livers from control mice and METTL3^Δhep^ mice (JAX) at 1 m and 3 m after birth (**E**), and quantification for Ki67 staining (**F**). (**G**, **H**) IHC of Ki67 in livers from 7-, 14- and 21- day-old control mice and METTL3^Δhep^ mice (GPT) (**G**), and quantification for Ki67 staining (**H**). (**I**, **J**) qRT-PCR analysis of the expression of cell cycle-related genes in the liver of METTL3^Δhep^ mice (JAX) (**I**) and METTL3^Δhep^ mice (GPT) (**J**).

These data from this study and the published study [17] provide strong evidence that hepatocyte-specific METTL3 homozygous knockout by Alb-iCre mice (GPT) results in liver injury, ALF and then postnatal lethality. Subsequently, we further explore the potential reasons why hepatocyte-specific METTL3 homozygous deletion by Alb-iCre mice (GPT), but not by Alb-Cre mice (JAX), leads to ALF and then postnatal lethality. To address that concern, based on mining three publicly available RNA-sequencing data deposited in the NCBI Gene Expression Omnibus (GEO) database under the accession No.: GSE198512 (postnatal lethality) [17], GSE197800 (postnatal survival) [16] and GSE176113 (postnatal survival) [20], we performed GO term enrichment analysis of differentially expressed genes (DEGs) in METTL3^Δhep^ mouse livers (JAX) versus control (GSE197800 [16] and GSE176113 [16, 20]) (Figure 8A, 8B) or METTL3^Δhep^ mouse livers (GPT) versus control (GSE198512) [17] (Figure 8C, 8D).

We wish to point out that GEO dataset (GSE198512) [17] is derived from METTL3^Δhep^ mice (GPT) which show postnatal lethality, while GEO datasets (both GSE197800 [16] and GSE176113 [20]) are derived from METTL3^Δhep^ mice (JAX) which show postnatal survival.

Top20 enrichment GO terms in “biological process” for up-regulated DEGs in METTL3^Δhep^ mouse livers (JAX) versus control (GSE197800 and GSE176113) [16, 20] includes mitotic cell cycle, mitotic cell cycle process, chromosome segregation, nuclear division, sister chromatid segregation, cell division, mitotic sister chromatid segregation, mitotic nuclear division, nuclear chromosome segregation, organelle fission, chromosome organization, microtubule cytoskeleton organization involved in mitosis, regulation of cell cycle process, microtubule cytoskeleton organization, spindle organization, mitotic spindle organization, regulation of chromosome separation, mitotic spindle checkpoint signaling, spindle checkpoint signaling, and regulation of mitotic sister chromatid segregation (Figure 8A, 8B, Supplementary Figure 5 and Supplementary Tables 7 and 8). Obviously, GO-term enrichment analyses based on the top20 significantly enriched GO terms in “biological process” for up-regulated DEGs revealed that upregulated genes in the livers of METTL3^Δhep^ mice (JAX) displaying postnatal survival are functionally enriched in terms associated with cell cycle, cell division, mitosis, microtubule cytoskeleton organization, spindle organization, chromatin segregation and organization, and nuclear division (Figure 8A, 8B, Supplementary Figure 5 and Supplementary Tables 7 and 8), consistent with the enhanced compensatory proliferation of the hepatocytes of METTL3^Δhep^ mice (JAX) observed *in vivo* (Figure 8E, 8F, 8I).

Unexpectedly, GO analysis illustrated that DEGs in the livers of 1 week-old and 2 week-old METTL3^Δhep^ mice (GPT) showing postnatal lethality are not functionally enriched in terms associated with cell cycle, cell division, mitosis, microtubule cytoskeleton organization, spindle organization, chromatin segregation and organization, and nuclear division (Figure 8C, 8D and Supplementary Tables 9 and 10), consistent with the loss of compensatory proliferation of the hepatocytes of METTL3^Δhep^ mice (GPT) observed *in vivo* (Figure 8G, 8H, 8J).

As the compensatory growth responses of METTL3^Δhep^ hepatocytes (JAX) to liver injury induced through hepatocyte-specific disruption of METTL3 by Alb-Cre mice (JAX), the increased proliferation of METTL3^Δhep^ (JAX) hepatocytes was evidenced by the marked increase of Ki67-positive hepatocytes (Figure 8E, 8F), accompanied by upregulation of cell-cycle-related genes (i.e., Ki67, CCNA2, CCNB1, CCNB2, CCND1 and CDK1) (Figure 8I). Conversely, surprisingly, as the compensatory proliferation responses of METTL3^Δhep^ hepatocytes (GPT) to severe liver injury induced through METTL3^Δhep^ (GPT), METTL3^Δhep^ (GPT) hepatocyte proliferation was not evidenced by the significant increase of Ki67-positive hepatocytes (Figure 8G, 8H), not accompanied by upregulation of cell-cycle-related genes (i.e., Ki67, CCNA2, CCNB1, CCNB2, CCND1 and CDK1) (Figure 8J).

Together, these aforementioned findings clearly demonstrate that ALF of METTL3^Δhep^ mice (GPT) might be mainly attributed to the loss of the compensatory growth responses of METTL3^Δhep^ hepatocytes (GPT) to severe liver injury induced by METTL3^Δhep^ (GPT), thereby leading to postnatal lethality of METTL3^Δhep^ mice (GPT).

## DISCUSSION

In 2019, to examine the functions of METTL3 in liver and underlying mechanisms, hepatocyte-specific METTL3 homozygous knockout (METTL3^Δhep^) was achieved by simultaneously crossing METTL3^fl/fl^ mice with Alb-iCre mice (GPT) or Alb-Cre mice (JAX), respectively. We observed that hepatocyte-specific METTL3 homozygous deficiency by Alb-iCre mice (GPT) or by Alb-Cre mice (JAX) causes liver injury, abnormal lipid accumulation and apoptosis. However, we were surprised to find that hepatocyte-specific METTL3 homozygous deletion by Alb-iCre mice (GPT), but not by Alb-Cre mice (JAX), resulted in ALF and then postnatal lethality. In the present study, we dissected the possible reasons why hepatocyte-specific METTL3 homozygous disruption in mice by Alb-iCre mice (GPT), but not by Alb-Cre mice (JAX), led to ALF and subsequently postnatal lethality.

In recent years, to examine the roles of METTL3 in the physiological and pathological processes of liver, and underlying mechanisms, the investigators around the world generated mice with hepatocyte-specific METTL3 homozygous knockout (METTL3^Δhep^) by crossing METTL3^fl/fl^ mice with Alb-Cre mice (JAX) (in this study and in other studies [16, 20–24, 30] or Alb-iCre mice (GPT) (in this study and in other study [17]) or with Alb-Cre-ETR^2^ mice [17] or by AAV-TBG-Cre injection [30, 37]. These results from this study and other studies revealed that hepatocyte-specific METTL3 homozygous knockout in mice by Alb-iCre mice (GPT) (in this study and in other study [17]) or by Alb-Cre mice (JAX) (in this study and in other studies [16, 20, 24, 30] resulted in liver injury, abnormal lipid accumulation and apoptosis.

Moreover, we generated mice with hepatocyte-specific METTL3 knockout by simultaneously crossing the same METTL3^fl/fl^ mice with Alb-iCre mice (GPT) or Alb-Cre mice (JAX), respectively, but we were surprised to observe that hepatocyte-specific METTL3 homozygous deletion by Alb-iCre mice (GPT), but not by Alb-Cre mice (JAX), led to ALF and then postnatal lethality, which is consistent with ALF and postnatal lethality phenotypes of METTL3^Δhep^ mice (GPT) in other study of Prof. Zhang’s lab [17]. More specifically, Prof. Zhang’s lab produced mice with hepatocyte-specific METTL3 knockout by simultaneously crossing the same METTL3^fl/fl^ mice with Alb-iCre mice (GPT) or Alb-Cre-ETR^2^ mice obtained from Beijing Biocytogen Co., Ltd. (Beijing, China) [17], and the results from Prof. Zhang’s lab demonstrated that hepatocyte-specific METTL3 homozygous disruption by Alb-iCre mice (GPT), but not by Alb-Cre-ETR^2^ mice (Beijing Biocytogen), resulted in postnatal lethality [17]. Additionally, our findings in this study clearly illustrated that METTL3^Δhep^ mice (GPT), which are about to die, exhibited the severe destruction of liver histological structure, indicating that METTL3^Δhep^ mice (GPT) nearly lose normal liver function, which subsequently contributed to ALF, followed by postnatal lethality. Furthermore, both our work and Prof. Zhang’s work revealed that METTL3^fl/wt^; Alb-iCre (GPT) mice and Alb-iCre mice (GPT) were fertile and survived without discernible defects in development, suggesting that one allele of METTL3 is enough to maintain the normal development and functions of liver in mice. Summarily, the aforementioned findings from this study and Prof. Zhang’s lab provide strong evidence that ALF and postnatal lethality are induced via hepatocyte-specific METTL3 homozygous disruption by Alb-iCre mice (GPT), but not via hepatocyte-specific METTL3 heterozygous knockout by Alb-iCre mice (GPT).

However, these results from this study and others labs [16, 17, 20, 24, 30] clearly exhibited that hepatocyte-specific METTL3 homozygous ablation in mice by Alb-Cre mice (JAX) [16, 20, 24, 30] induced liver injury, but didn’t cause ALF and then postnatal lethality because homozygous METTL3 knockout in mice by Alb-Cre mice (JAX) didn’t lead to the severe destruction of liver histological structure of METTL3^Δhep^ mice (JAX), thereby METTL3^Δhep^ mice (JAX) still had well-maintained liver function. In addition, hepatocyte-specific METTL3 homozygous disruption in mice by Alb-Cre mice (JAX) [21–23] or by AAV-TBG-Cre injection [30, 37] didn’t also lead to postnatal lethality. Collectively, the above-mentioned data from this study and other labs [16, 20–24, 30, 37] provide strong evidence that ALF and postnatal lethality are not induced via heterozygous or homozygous METTL3 disruption by Alb-Cre mice (JAX).

Additionally, our other study findings demonstrated that hepatocyte-specific METTL14 homozygous ablation by Alb-iCre mice (GPT), but not by Alb-Cre mice (JAX), also resulted in postnatal lethality (Supplementary Figure 2), whereas METTL14^fl/wt^; Alb-iCre (GPT) mice was fertile and survived without discernible defects in development (Supplementary Figure 2), suggesting that one allele of METTL14 is enough to maintain the normal development and functions of liver in mice. These results from two other labs clearly showed that hepatocyte-specific METTL14 homozygous disruption by Alb-Cre mice (JAX) didn’t induce postnatal lethality [38, 39], which is consistent with postnatal survival phenotype of METTL14^Δhep^ mice (JAX) observed in our other study (Supplementary Figure 4).

Collectively, it is very evident that postnatal lethality caused by METTL3 or METTL14 homozygous knockout via Alb-iCre mice (GPT) might be closely related with Alb-iCre mice (GPT), but not the real functions of METTL3 or METTL14.

Next, we analyzed the possible reasons for ALF and then postnatal lethality of METTL3^Δhep^ mice (GPT). As the enhanced compensatory proliferation responses of hepatocytes to liver injury induced through METTL3^Δhep^ (JAX), these results from this study and other studies [16, 20, 30] revealed that the increased growth of METTL3^Δhep^ (JAX) hepatocytes was evidenced by the significant increase of Ki67-positive hepatocytes, accompanied by upregulation of cell-cycle-related genes. In addition, GO analysis based on the top20 significantly enriched GO terms in "biological process" for up-regulated DEGs obviously revealed that upregulated genes in the livers of METTL3^Δhep^ mice (JAX) displaying postnatal survival are functionally enriched in terms associated with cell cycle, cell division, mitosis, microtubule cytoskeleton organization, spindle organization, chromatin segregation and organization, and nuclear division [16], consistent with the enhanced compensatory proliferation capacity of METTL3^Δhep^ hepatocytes (JAX) observed in this study and in other studies from other labs [16, 20, 30].

However, surprisingly, as the compensatory proliferation responses of hepatocytes to liver injury, we unexpectedly found that the proliferation of METTL3^Δhep^ hepatocytes (GPT) was not evidenced by the dramatic increase of Ki67-positive hepatocytes, not accompanied by upregulation of cell-cycle-related genes. Additionally, GO analysis illustrated that upregulated genes in METTL3^Δhep^ livers (GPT), unlike METTL3^Δhep^ livers (JAX), are not functionally enriched in terms associated with cell cycle, cell division, mitosis, microtubule cytoskeleton organization, spindle organization, chromatin segregation and organization, and nuclear division, consistent with the loss of compensatory proliferation of METTL3^Δhep^ hepatocytes (GPT) observed *in vivo*. Therefore, obviously, these aforementioned findings clearly demonstrate that ALF of METTL3^Δhep^ mice (GPT) is attributed to, at least partially, the loss of the compensatory growth capacity of METTL3^Δhep^ hepatocytes (GPT), thereby leading to postnatal lethality of METTL3^Δhep^ mice (GPT). However, the causes of the loss of the compensatory growth capacity of METTL3^Δhep^ hepatocytes (GPT) are still unknown.

Until now, Alb-Cre mice (JAX) [40] are frequently and successfully employed to delete loxP-flanked DNA fragment for conditional gene knockout or overexpression in the hepatocytes of mice. In Alb-Cre mice (JAX), Cre recombinase was engineered to be expressed in mouse hepatocytes under the control of mouse albumin enhancer/promoter (Alb) [40], while the Alb-Cre transgene inserted in reverse orientation on chromosome 13 causing an 4 bp deletion in Speer6-ps1 (spermatogenesis associated glutamate (E)-rich protein 6, pseudogene 1) [40].

Alb-iCre mice (GPT) are generated via inserting Alb-promoter-iCre transgene into the H11 genomic locus between Eif4enif1 and Drg1 genes by CRISPR/Cas9-based knockin technology. Alb-iCre mouse strain (GPT) shows normal development and is fertile, with no apparent abnormalities in general appearance or behavior.

As the insertion site of Alb-promoter-iCre transgene in Alb-iCre mice (GPT) is located on chromosome 11, please avoid using Alb-iCre mice (GPT) to knockout genes of interest located on chromosome 11. METTL3 and METTL14 are located on chromosome 14 (Chromosome 14: 52,532,298-52,542,585 reverse strand) and chromosome 3 (Chromosome 3: 123,161,946-123,179,757 reverse strand), respectively. Therefore, theoretically speaking, Alb-iCre mice (GPT) are suitable to knockout METTL3 or METTL14 gene.

Up to now, Alb-iCre mice (GPT) have been used to knockout the following genes in mice, FGF4 [41], PP2Acα [42], METTL3 [17], and METTL3 and METTL14 (this study). Both METTL3^Δhep^ mice (GPT) (this study and other lab study [17]) and METTL14^Δhep^ mice (GPT) (this study) exhibit postnatal lethality, whereas both FGF4^Δhep^ mice (GPT) [41] and PP2Acα^Δhep^ mice (GPT) [42] are not reported to show postnatal lethality, which makes it very unclear why postnatal lethality is induced via hepatocyte-specific METTL3 or METTL14 homozygous disruption by Alb-iCre mice (GPT), but not induced via hepatocyte-specific FGF4 [41] or PP2Acα [42] homozygous knockout by Alb-iCre mice (GPT).

## CONCLUSION

Although hepatocyte-specific METTL3 homozygous deficiency by Alb-iCre mice (GPT) or by Alb-Cre mice (JAX) leads to the following same phenotypes: liver injury, abnormal lipid accumulation and apoptosis observed in this study and other studies [16, 17, 20, 24, 30], these aforementioned findings from this study and other labs provide very strong evidence that these phenotypes (i.e., ALF and postnatal lethality) of METTL3^Δhep^ mice (GPT) might be not the real functions of METTL3, and closely related with Alb-iCre mice (GPT). In addition, based on our findings, those from other labs and our in-depth discussion, we remind investigators to apply Alb-iCre mice (GPT) with caution to knockout genes in hepatocytes *in vivo*.

## MATERIALS AND METHODS

### Mice

The wildtype C57BL/6J mice were purchased from the Laboratory Animal Management Center, Southern Medical University and the Guangdong Medical Laboratory Animal Center. The Albumin-Cre transgenic mice (Alb-Cre mice (JAX)) (B6.Cg-*Speer6-ps1*^*Tg(Alb-cre)21Mgn/J*^; https://www.jax.org/strain/003574) were obtained from Model Animal Research Center of Nanjing University. Alb-iCre mice (GPT) (C57BL/6JGpt-H11^em1Cin(Alb-iCre)^/Gpt; Strain No. T003814; https://www.gempharmatech.com/shop/detail/6902.html) were purchased from the GemPharmatech Co., Ltd., (Nanjing, China).

METTL3^fl/wt^ mice were purchased from the Shanghai BRL Medicine Technology Co., Ltd., (Shanghai, China). The conditional mutant alleles for METTL3 were generated by the CRISPR/Cas9 technology. METTL3^fl/fl^ mice, in which exon 4 of the METTL3 allele is flanked by loxP sites, were obtained by mating METTL3^fl/wt^ mice and METTL3^fl/wt^ mice. METTL3^fl/fl^/Alb-Cre mice (hereinafter referred to as METTL3^Δhep^ (JAX)) were generated by crossing METTL3^fl/fl^ mice with Alb-Cre mice (JAX), while METTL3^fl/fl^/Alb-iCre mice (hereinafter referred to as METTL3^Δhep^ (GPT)) were generated by crossing METTL3^fl/fl^ mice with Alb-iCre mice (GPT).

All mice described above were maintained on the C57BL/6J (B6) background. All animal care and experimentation were performed according to the Study and Ethical Guidelines for Animal Care, handling and termination established by the Subcommittee of Southern Medical University on laboratory animal care. The presented work was approved by the ethical committee of Southern Medical University and is covered by Chinese animal husbandry legislation.

### PCR-based genotyping assay

Mice were genotyped by PCR with mouse tail or toe genomic DNA. Genotypes were determined by PCR using primers specific for iCre (GPT): 5′-CCTGCTGTCCATTCCTTATTCCAT-3′ (forward), and 5′-ATATCCCCTTGTTCCCTTTCTGC-3′ (reverse); specific for Cre (JAX): 5′-ATCCGAAAAGAAAACGTTGA-3′ (forward), and 5′-ATCCAGGTTACGGATATAGT-3′ (reverse); specific for METTL3: 5′-TAGTGCTGTGCCTTTCTTAG-3′ (METTL3-L-LoxP-F), and 5′-TTAAACTGACTGCCTCCATA-3′ (METTL3-L-LoxP-R); and specific for Myo: 5′-TTACGTCCATCGTGGACAGC-3′ (forward), and 5′-TGGGCTGGGTGTTAGCCTTA-3′ (reverse). The genomic DNA from wild-type (WT) mice was employed as a negative control for each PCR test.

Furthermore, to assess the knockout efficiency of METTL3 in liver, the main organ and tissue genomic DNA, including the liver, was subjected to PCR using the following primer pairs to amplify 338bp METTL3 mutant fragment, F1: GTGCTGTGCCTTTCTTAG, R1: AGCGTCACTGGCTTTCAT, and R2: TTCTTGTTCTCCCCCAAT. A 338bp band can be only observed in tissues with successful deletion of METTL3.

### RNA extraction, reverse transcription (RT) and quantitative real-time PCR (qRT-PCR)

To quantitate mRNA expression, total RNA was extracted from the mouse livers using TRIzol reagent (TaKaRa), and reversely transcribed into cDNA with the PrimeScript RT reagent Kit (TaKaRa). qRT-PCR was performed using the SYBR Green qPCR Master Mix (TaKaRa) on a LightCycler 96 system (Roche) following the manufacturer’s instructions. To measure the levels of the indicated mRNAs, GAPDH was used as endogenous control. All primers used in this study are listed in Supplementary Table 11. Relative gene expression was analyzed using the 2^ΔΔCt^ method with Gapdh as the internal control.

### Western blotting

Western blot analysis was performed as previously described [3, 4, 30, 43–46]. GAPDH was used as loading control. The primary antibodies used in this study are listed in Supplementary Table 12.

### Histological analysis, immunohistochemistry (IHC) and Oil Red O staining

Formalin-fixed, paraffin-embedded mouse liver tissues were cut into 4 μm sections and subjected to hematoxylin and eosin staining (H&E staining) according to standard procedures, as described previously [3, 4, 44, 46–48]. Immunohistochemical staining was performed as previously described [3, 4, 44, 46–48]. The antibodies used in the study and the experimental conditions are summarized in the Supplementary Table 12.

According to the manufacturer's instructions, liver lipid accumulation was detected using a Modified Oil Red O staining kit (Catalog No. C0158S; Beyotime, Beijing, China), as previously described [4]. Fresh liver tissues were embedded in Tissue-Tek OCT compound and cut into 8 μm sections for staining with oil Red O for detection.

### Measurement of serum parameters

The serum supernatant was obtained by centrifugation at 800 g for 10 min. The serum levels of ALT (Alanine transaminase) and AST (Aspartate aminotransferase) were detected using the Beckman automatic biochemical analyzer AU680 (Beckman Coulter, Brea, CA, USA) according to the manufacturer’s instructions.

### TUNEL assay

To detect oligonucleosomal DNA strand breaks in individual apoptotic cells, the formalin-fixed, paraffin-embedded liver sections were subjected to terminal deoxynucleotidyl transferase-mediated 2′-deoxyuridine 5′-triphosphate nick-end labeling (TUNEL) staining using a TUNEL kit according to the manufacturer’s instructions (KeyGEN, KGA704, Nanjing, China), as described previously [49]. The number of TUNEL-positive cells was counted at 100× magnification in six randomly selected fields from each liver sample. The total number of cells in each of the six fields was demonstrated.

### Statistical analysis

The data were presented as means ± SD. Statistical analysis was performed using the SPSS 16.0 software package and GraphPad 8.1 software. Two-tailed Student’s *t*-test was employed to compare data between two independent groups. Statistical significance was set at ^*^P < 0.05, ^**^P < 0.01 and ^***^P < 0.001.

### Data availability

The RNA-seq data sets reported in the studies of the labs of Prof. Qi Zhang (The Third Affiliated Hospital, Sun Yat-sen University, Guangzhou, China) [17], Prof. Detian Yuan (School of Basic Medical Sciences, Cheeloo College of Medicine, Shandong University, Jinan, China) [16] and Prof. Kalpana Ghoshal (College of Medicine, The Ohio State University, Columbus, Ohio) [20] have been deposited in the NCBI Gene Expression Omnibus (GEO) database under the accession No. GSE198512, GSE197800 and GSE176113, respectively.

## Supplementary Materials

Supplementary Figures

Supplementary Tables 1, 3 and 11-12

Supplementary Tables 2 and 4-10
