# Supplementary material for: Hepatocyte-specific METTL3 ablation by Alb-iCre mice (GPT), but not by Alb-Cre mice (JAX), resulted in acute liver failure (ALF) and postnatal lethality
Source: Aging (Albany NY). 2024 Apr 22;16(8):7217–48. doi: 10.18632/aging.205753 (PMC11087113; doi:10.18632/aging.205753)
Supplement: Supplementary Figures [file aging-16-205753-s001.pdf]

SUPPLEMENTARY FIGURES

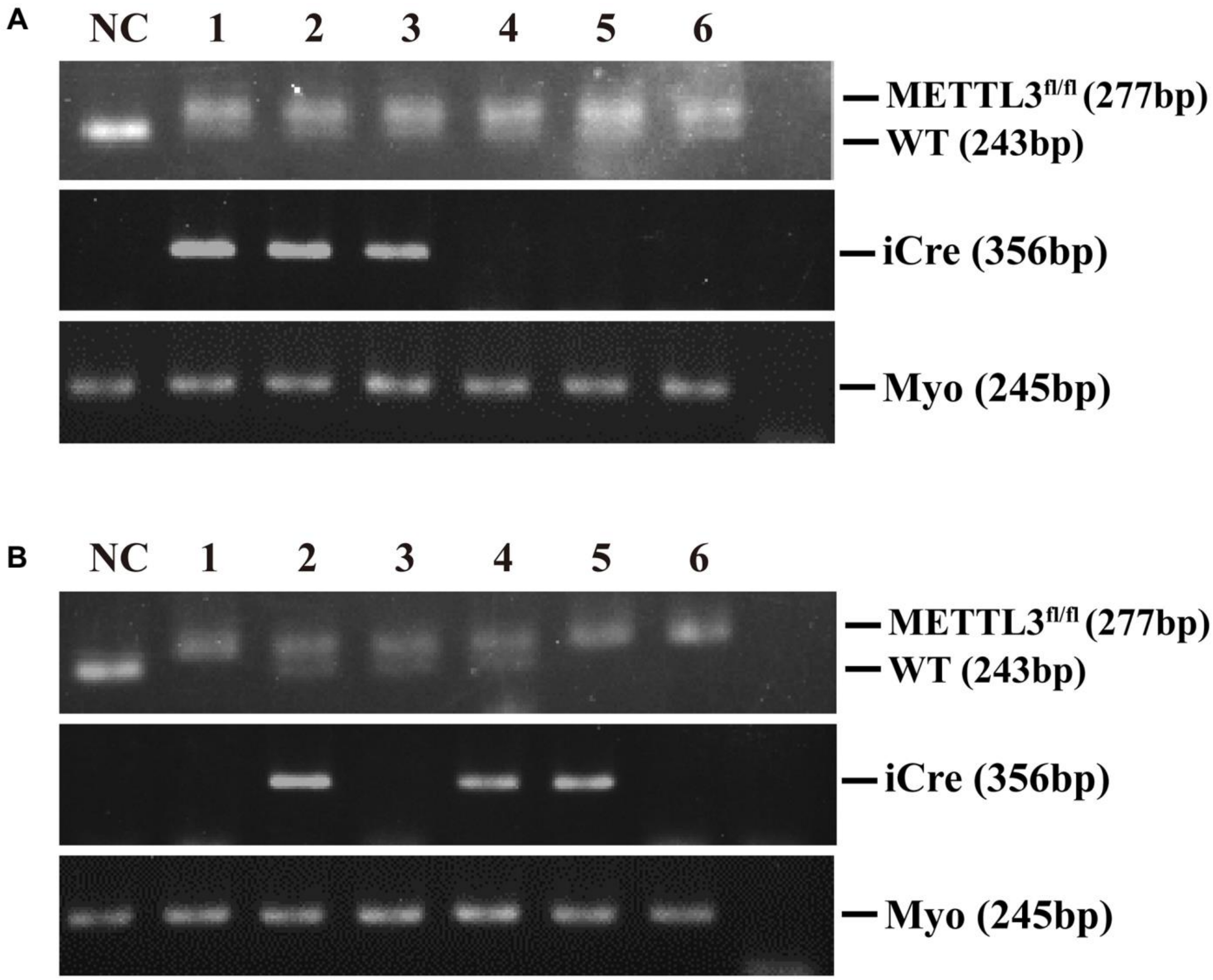

Supplementary Figure 1. PCR-based genotyping of hepatocyte-specific METTL3 homozygous knockout (METTL3$^{\Delta hep}$) mice by Alb-iCre mice (GPT). (**A**) PCR-based genotyping displays the offspring with indicated genotypes from intercrossing METTL3$^{fl/fl}$ mice and Alb-iCre (GPT) mice. (**B**) PCR-based genotyping exhibits the offspring with indicated genotypes from intercrossing METTL3$^{fl/fl}$ mice and METTL3$^{fl/wt}$; Alb-iCre (GPT) mice.

A

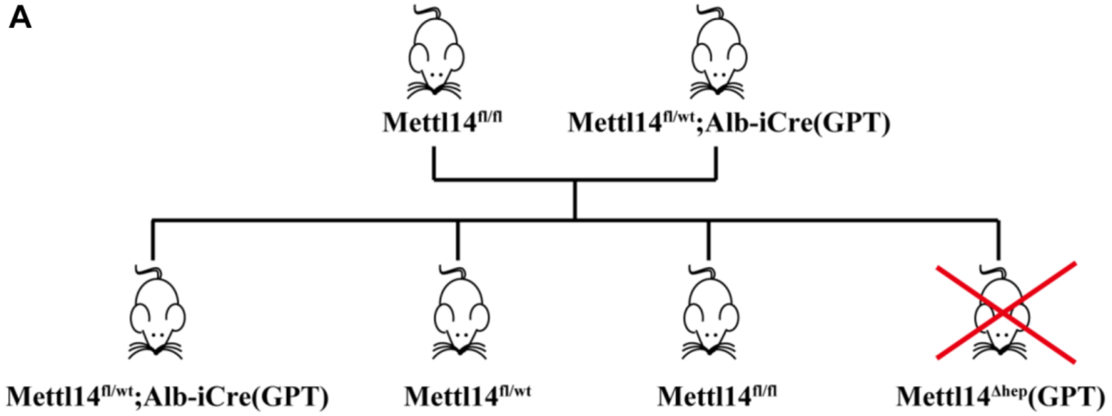

B

Genotype	Number	Ratio (%)
Mettl14$^{fl/wt}$	15	38.46
Mettl14$^{fl/wt}$;Alb-iCre(GPT)	12	20.77
Mettl14$^{fl/fl}$	12	30.77
Mettl14$^{\Delta hep}$(GPT)	0	0
Total	39	

C

Genotype	Number	Ratio (%)
Mettl14$^{fl/wt}$	6	28.57
Mettl14$^{fl/wt}$;Alb-iCre(GPT)	6	28.57
Mettl14$^{fl/fl}$	5	23.81
Mettl14$^{\Delta hep}$(GPT)	4	19.05
Total	21	

Supplementary Figure 2. Hepatocyte-specific METTL14 homozygous ablation in mice by Alb-iCre mice (GPT) results in postnatal lethality. (**A**) A schematic representation of the offspring with indicated genotypes from intercrossing METTL14$^{fl/fl}$ and METTL14$^{fl/wt}$; Alb-iCre (GPT) mice. (**B**) PCR-based genotyping during the late postnatal period displays the number of offspring with indicated genotypes from intercrossing METTL14$^{fl/fl}$ mice and METTL14$^{fl/wt}$; Alb-iCre (GPT) mice. (**C**) PCR-based genotyping during the early postnatal period exhibits the number of offspring with indicated genotypes from intercrossing METTL14$^{fl/fl}$ mice and METTL14$^{fl/wt}$; Alb-iCre (GPT) mice.

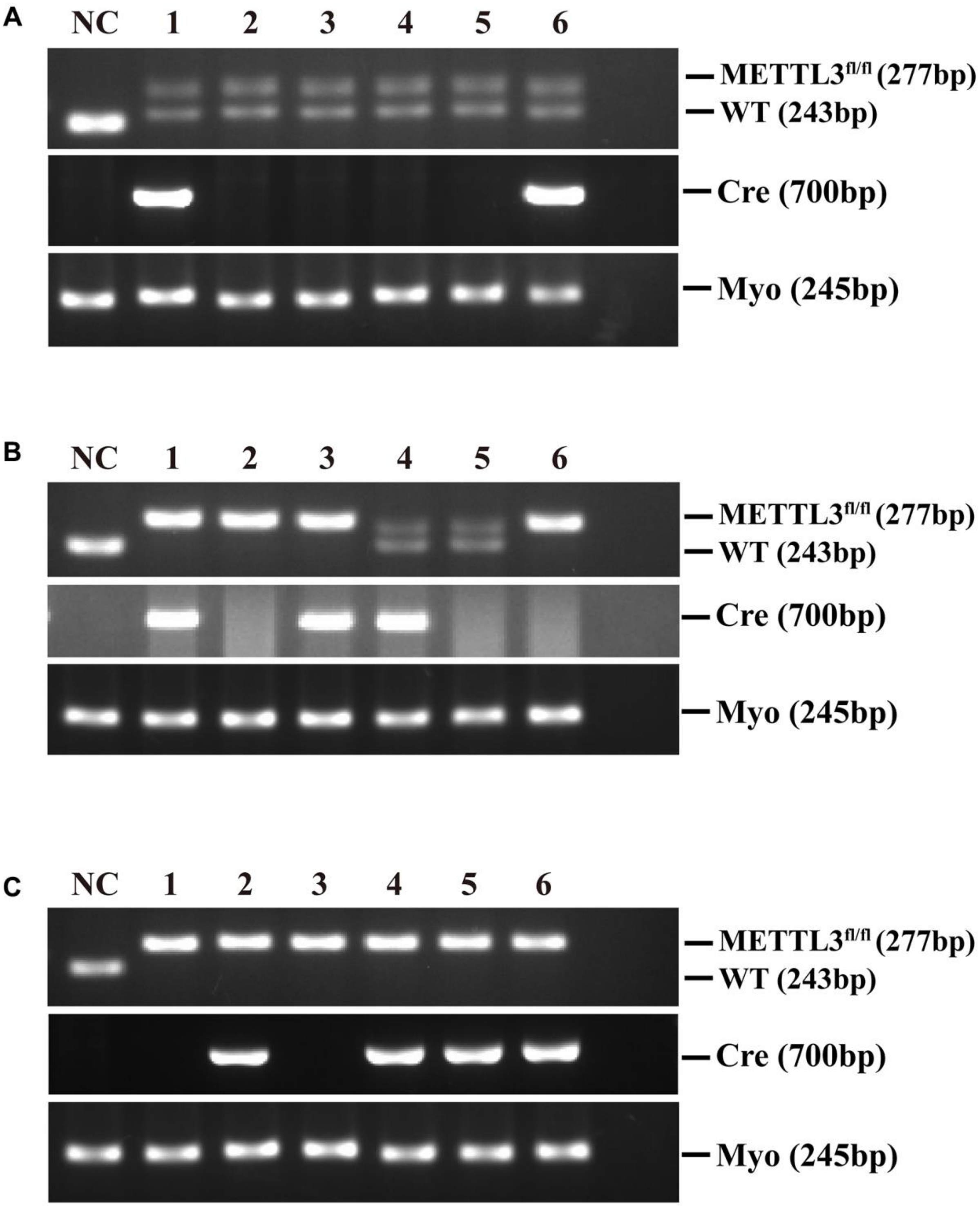

Supplementary Figure 3. PCR-based genotyping of hepatocyte-specific METTL3 homozygous knockout (METTL3$^{\Delta hep}$) mice by Alb-Cre mice (JAX). (**A**) PCR-based genotyping displays the offspring with indicated genotypes from intercrossing METTL3$^{fl/fl}$ mice and Alb-Cre (JAX) mice. (**B**) PCR-based genotyping exhibits the offspring with indicated genotypes from intercrossing METTL3$^{fl/fl}$ mice and METTL3$^{fl/wt}$; Alb-Cre (JAX) mice. (**C**) PCR-based genotyping exhibits the offspring with indicated genotypes from intercrossing METTL3$^{fl/fl}$ mice and METTL3$^{fl/fl}$; Alb-Cre (JAX) mice.

A

Genotype	Number	Ratio (%)
Control	80	44.69
Mettl14$^{\Delta hep}$(JAX)	99	55.31
Total	179	

B

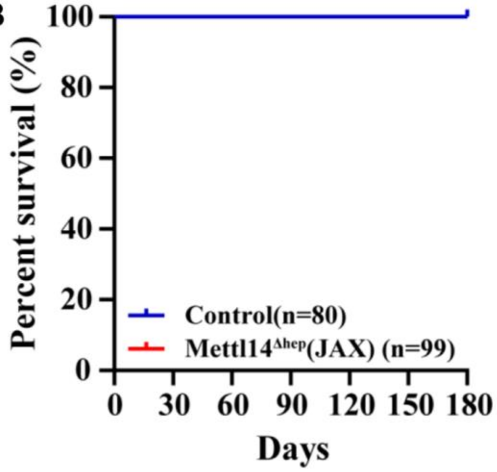

Supplementary Figure 4. Hepatocyte-specific ablation of METTL14 in mice by Alb-Cre mice (JAX) didn't lead to postnatal lethality. (**A**) PCR-based genotyping during the late postnatal period shows the presence of offspring with the genotype (i.e., METTL14$^{fl/fl}$; Alb-Cre (JAX): referred to as METTL14$^{\Delta hep}$ (JAX)) from intercrossing METTL14$^{fl/fl}$ mice and METTL14$^{fl/fl}$; Alb-Cre (JAX) mice. (**B**) Survival curves of control and METTL14$^{\Delta hep}$ (JAX) mice (*n* = 80–99 for each group).

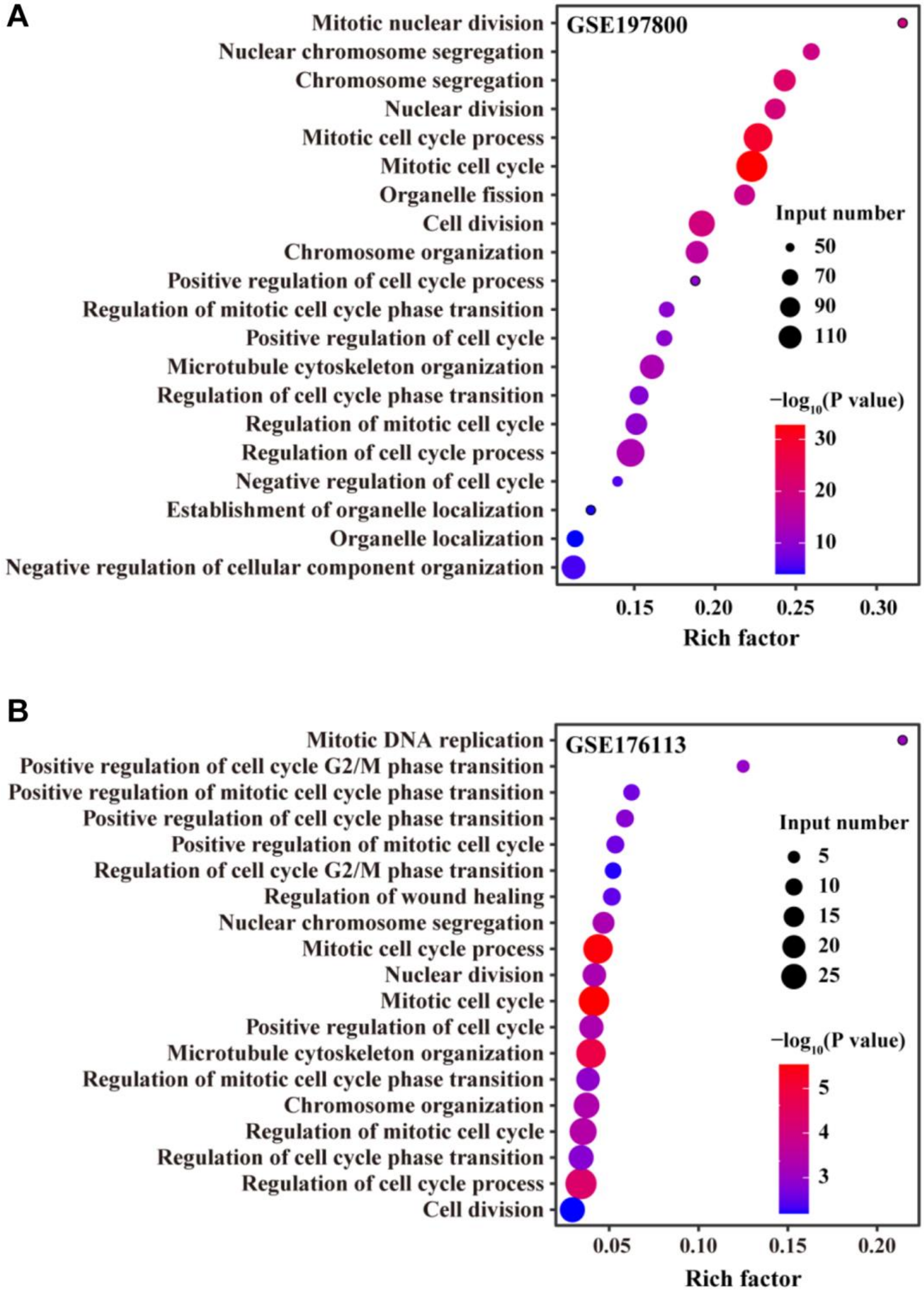

Supplementary Figure 5. GO term enrichment analyses of up-regulated DEGs (from RNA-seq data deposited in GEO under accession number GSE197800 (**A**) and GSE176113 (**B**)) in the liver of control and METTL3$^{\Delta hep}$ (JAX) mice.
