# Supplementary material for: Hepatocyte-specific METTL3 ablation by Alb-iCre mice (GPT), but not by Alb-Cre mice (JAX), resulted in acute liver failure (ALF) and postnatal lethality
Source: Aging (Albany NY). 2024 Apr 22;16(8):7217–48. doi: 10.18632/aging.205753 (PMC11087113; doi:10.18632/aging.205753)
Supplement: Supplementary Tables 1, 3 and 11-12 [file aging-16-205753-s002.pdf]

SUPPLEMENTARY TABLES

Supplementary Table 1. Hierarchical clustering of differentially expressed genes (DEG) related to apoptosis in the liver of control mice and METTL3$^{\Delta hep}$ mice (GPT) at 1 week after birth from RNA-seq data deposited in GEO under accession number GSE198512.

Gene	WT1	WT2	KO1	KO2	log2FC (1 week)	*P*-value (1 week)	FDR (1 week)
Scn2a	0	0.01553	0.28925	0.09587	4.52098	0.0012	0.02168
Eda2r	0.04427	0.0146	0.42149	0.61588	4.06888	8.6E-05	0.00268
Mmp12	0.04702	0	0.30327	0.19146	3.31959	0.01667	0.14327
Ifi27l2b	1.21898	1.07919	7.74376	13.976	3.16893	1.2E-11	2.1E-09
Phlda3	1.9058	1.64398	16.614	16.4651	3.13858	1.1E-22	6.9E-20
Fa2h	0.11256	0.16706	0.51854	1.0312	2.39486	0.00879	0.09171
Degs1l	0.44335	0.29246	2.34884	1.42915	2.27339	0.00069	0.01429
Aen	1.23099	1.61004	6.70089	7.38882	2.22698	1.5E-16	5.9E-14
Tnfrsf10b	0.5294	0.34922	1.78852	2.17806	2.09844	3.1E-05	0.00116
Rnf186	0.39301	1.11106	2.01745	4.57211	2.05332	0.00267	0.03867
Zpr1	11.9614	10.0916	53.5242	43.1306	2.04683	5.3E-26	4.1E-23
Bmp7	0.28661	0.49156	1.19714	2.06172	1.98764	0.00024	0.00622
Isg15	11.2235	11.7478	44.5263	49.0988	1.94582	1.4E-14	3.9E-12
Ifi206	0.23014	0.36435	0.91179	1.42905	1.89755	0.00339	0.04612
Ifi27l2a	9.0873	8.99165	35.1658	34.6889	1.86729	6.5E-09	6.6E-07
Ifi213	1.67934	0.8982	4.16098	4.96671	1.75028	2.7E-06	0.00015
Ifi211	0.94284	0.5442	2.53373	2.63934	1.72115	0.00187	0.02988
Ifit1	1.95375	2.0638	5.05983	8.14336	1.64154	2E-06	0.00011
Tgtp2	0.32442	0.41977	1.12665	1.24474	1.5885	0.01058	0.10415
Ifi208	0.51596	0.37129	1.46939	1.31305	1.56742	0.00511	0.06246
Ifit3	5.62597	5.7258	18.9572	16.1581	1.54371	9.1E-10	1.1E-07
Bax	19.3159	20.1218	63.5529	57.6248	1.53475	6.9E-14	1.7E-11
Ifit2	1.2673	1.2903	3.43527	4.37491	1.53168	4.4E-06	0.00022
Dhx58	2.09324	1.63971	5.78598	5.29742	1.48817	1E-05	0.00044
Oas1g	1.38332	0.63174	2.41839	3.43009	1.46695	0.00484	0.05975
Elovl7	0.33201	0.32851	0.94991	0.96054	1.44975	0.01063	0.1046
St8sia3	0.16744	0.19881	0.55539	0.47724	1.40904	0.02147	0.17002
Stk11	36.2009	31.8799	95.4247	92.1361	1.38026	1.7E-16	6.3E-14
Tnfrsf12a	14.5636	14.1941	40.1213	38.026	1.35899	4.2E-09	4.4E-07
Bst2	44.4471	40.2121	115.107	113.833	1.35354	1.7E-12	3.3E-10
Irf7	6.125	7.81658	19.403	17.9777	1.33654	1.5E-09	1.7E-07
Oas1a	1.59964	1.50918	4.97001	3.25669	1.31544	0.00189	0.03003
Trim6	0.5497	0.43513	1.35062	1.21239	1.29768	0.00952	0.09716
Ifi204	4.42854	2.8407	9.79282	8.33205	1.23695	3.7E-05	0.00134
Ythdf2	3.48156	4.10029	8.60634	9.97516	1.21173	6.5E-07	4.2E-05
Gal3st1	4.62764	4.20966	13.0664	8.16772	1.17516	0.00012	0.00358
Xaf1	3.42923	2.91222	8.18511	6.81662	1.1582	4.8E-05	0.00167
Xkr6	1.86888	1.07737	3.14441	3.54015	1.10647	0.00132	0.02314
Oas3	0.62398	0.92612	1.88902	1.64844	1.10198	0.00614	0.07132
Smpd1	7.23914	6.55884	17.4383	13.8503	1.09555	2.9E-06	0.00016
Cdc34	37.6652	40.1187	79.7164	93.3154	1.07348	5.7E-08	4.9E-06

Gbp3	4.3141	4.65675	11.0574	8.54349	1.03993	6.4E-05	0.00213
Cln6	3.83029	4.50261	11.5828	6.49837	1.02362	0.00148	0.02537

Supplementary Table 3. Heatmap (RNA-seq results deposited in NCBI GEO under the accession number GSE198512) depicts the differential expression of hepatic lipid metabolism-related genes in the liver of control mice and METTL3$^{\Delta hep}$ mice (GPT) at 2 week after birth.

Gene	WT1	WT2	KO1	KO2	log2FC (2 weeks)	*P*-value (2 weeks)	FDR (2 weeks)
Ucp3	0.238358	0.208127	8.833701	7.629526	4.87111	1.12E-19	5.16E-17
Fa2h	0.03345	0.068151	2.081482	1.476251	4.792228	6.1E-06	0.000154
Acot1	0.223307	0.227482	7.465077	5.631563	4.520973	5.61E-17	1.66E-14
Plin4	0.854425	0.590103	9.125791	8.554477	3.282266	1.42E-24	9.14E-22
Cyp4a14	46.62122	66.61264	424.1761	402.966	2.545859	7.78E-27	6.06E-24
Cox6a1	125.0357	124.3606	827.6662	787.7966	2.368635	1.03E-27	1.02E-24
Ephx1	27.53036	26.92334	172.3829	167.3779	2.315567	7.05E-25	4.74E-22
Elovl7	0.124631	0.169281	0.873845	0.79332	2.177054	0.004941	0.033264
Aldh1b1	8.63316	10.82695	55.12664	53.41392	2.156227	5.05E-18	1.87E-15
Gstp1	31.93338	38.1392	169.6623	165.6048	1.934893	2.37E-15	5.39E-13
St3gal2	0.758482	0.772664	3.772512	3.122116	1.83633	2.95E-06	8.25E-05
Lpin1	4.764546	2.38878	16.06183	15.6806	1.817315	1.72E-08	9.35E-07
Cox8a	85.80433	103.6129	463.9461	342.1794	1.750053	5.72E-14	1.11E-11
Uqcr10	92.51009	91.57587	413.1307	315.5849	1.645475	4.94E-13	8.13E-11
Cyp4a32	9.915687	13.81963	51.04447	38.97994	1.587408	5.8E-09	3.67E-07
Ndufs4	8.885538	10.15189	39.75545	29.99852	1.534864	8.05E-09	4.84E-07
Pla2g12a	2.117361	2.10019	7.422914	6.715244	1.415196	2.67E-05	0.000524
Aig1	1.297387	0.952363	4.130259	3.303339	1.384628	0.00025	0.003247
Cpt2	14.5137	15.39368	55.28562	43.27706	1.383201	4.08E-09	2.8E-07
Ndufa1	43.96642	53.09771	175.7555	139.6357	1.365025	1.39E-07	5.96E-06
Abhd5	2.191525	2.60907	8.122598	6.991736	1.323826	4.42E-05	0.000791
Uqcrfs1	43.54386	55.26069	171.0383	137.133	1.307011	1.68E-08	9.16E-07
Cln6	2.257103	2.378594	7.230674	6.572622	1.24507	0.000847	0.008632
Uqcr11	93.96624	81.68761	265.6622	238.901	1.190735	3.23E-07	1.2E-05
Ndufb6	17.96163	20.8746	60.86244	50.7786	1.190636	3.04E-05	0.000585
Pigh	0.755678	0.532944	2.012382	1.652725	1.169531	0.038167	0.144255
Slc27a4	1.295387	1.319609	3.910555	3.315697	1.133227	0.002177	0.018207
Glul	103.8083	103.6204	293.8769	272.5464	1.121003	9.75E-08	4.34E-06
Por	47.90822	40.49286	121.7526	116.4795	1.101938	1.33E-06	4.11E-05
Ndufa6	111.639	120.3985	332.1489	266.3101	1.031083	4.19E-06	0.000111
Plbd1	11.2196	11.42939	29.46423	27.81046	1.011264	7.8E-05	0.001259
Mif	80.02286	80.52849	220.6324	185.5917	1.005428	1.16E-05	0.000263
Abca8b	23.3126	22.53592	15.18619	13.53362	−1.00581	4.68E-06	0.000122
Pik3cd	3.591082	3.813533	1.915132	2.487126	−1.06062	0.001792	0.015637
Gpat2	3.013291	3.489691	1.66797	2.126703	−1.08843	0.010319	0.056554
Mapk12	12.31162	11.91249	5.878602	7.715077	−1.14421	0.000438	0.005186
Cycs	5.525113	6.743234	2.503077	4.281489	−1.1492	0.002193	0.018273
Myo5a	0.377536	0.290261	0.181051	0.157188	−1.31634	0.022796	0.100931

Cyp3a44	21.96516	21.38043	10.79845	7.067439	−1.62595	4.67E-09	3.14E-07
Insig1	15.39186	12.68857	5.36348	5.613553	−1.67947	5.83E-09	3.67E-07
Sult2a8	37.30261	38.43599	14.42051	10.10193	−1.97023	2.1E-15	4.85E-13
Cyp2a22	210.8488	240.1306	54.05589	54.44093	−2.37754	1.64E-27	1.51E-24
Gramd1c	25.38095	26.94701	4.507379	4.304201	−2.89678	9.74E-32	1.31E-28
Sult2a6	4.337	3.534477	0.76022	0.515323	−2.96958	2.23E-05	0.000451
Cyp2b13	28.05246	25.31623	6.818662	1.622453	−3.05736	0.00027	0.003446
Sult2a5	20.95882	17.9411	0.838131	4.125752	−3.22379	0.002984	0.023034
Cyp3a41b	0.888497	0.493697	0.035396	0.137106	−3.29321	0.005464	0.035603
Cyp3a16	40.57036	39.70919	3.67397	2.944363	−3.93658	5.26E-47	2.6E-43
Rdh16f2	39.80057	38.73235	3.811675	2.628526	−3.95037	1.28E-39	4.73E-36

Supplementary Table 11. Primers for qRT-PCR analysis of mouse gene expression.

Gene	Forward Primer (5′–3′)	Reverse Primer (5′–3′)
Abcd3	GGCCTGCACGGTAAGAAAAGT	CCGCAATAAGTAACAAGTAGCCT
Abcg8	CTGTGGAATGGGACTGTACTTC	GTTGGACTGACCACTGTAGGT
Acaa1a	TCTCCAGGACGTGAGGCTAAA	CGCTCAGAAATTGGGCGATG
Acaa2	CTGCTACGAGGTGTGTTCATC	AGCTCTGCATGACATTGCCC
Acad11	TGACACCGTGGAAGTGCTAC	CCCGGCAAGTGCTGATTCA
Adh7	ATGGGCACCGCTGGAAAAG	TAACACGGACTTCCTTAGCCT
Ang	CCAGGCCCGTTGTTCTTGAT	GGAAGGGAGACTTGCTCATTC
Apoa2	TGGTCGCACTGCTGGTAAC	TTTGCCATATTCAGTCATGCTCT
Apoa5	TCCTCGCAGTGTTCGCAAG	CGAAGCTGCCTTTCAGGTTCT
Apof	ATAGCCTCCGACTCATCCTGA	TCTGCATCTGGTATCCCAACTT
Avpr1a	TGAGTTTCGTTCTGAGCATACC	CCCAGCAATCTTGGGCTTTG
Bax	GTGAGCGGCTGCTTGTCTGG	CTTCCAGATGGTGAGCGAGG
Bcl2	GAGGATTGTGGCCTTCTTTG	CGTTATCCTGGATCCAGGTG
Bcl2a1	GGCTGAGCACTACCTTCAGTA	TGGCGGTATCTATGGATTCCAC
Bdh2	CGACTGGACGGCAAAGTTATT	CCTGGAGTTTGGACTCGTTGA
Bik	CCTGCATCGGCGATGAGATG	CTCTGACACCTGTCCGGCTG
Ccna2	TGAATCACCACATGCTAT	TAACCTCCATTTCCCTAAG
Ccnb1	CTCTGTAGTGAATATGTG	CATCTGAACCTGTATTAG
Ccnb2	CTCCATGTAGCCTGTGTAA	TCTTGCCTGTCTCAGAAG
Ccnd1	TGCCATCCATGCGGAAA	AGCGGGAAGAACTCCTCTTC
Cdk1	GACAATCAGATTAAGAAGA	AACTATACAAGACAGGAA
Ces1b	TACCTCCCCTGTTTTCCGAAG	GATGCTCCGCCTGTCATCAAT
Ces1d	ATGCGCCTCTACCCTCTGATA	AGCAAATCTCAAGGAGCCAAG
Ces1e	CAACTTCTGGAATTGATTGGGGA	GGGCTCCGGCATCTCTATG
Cpt1a	CTCCGCCTGAGCCATGAAG	CACCAGTGATGATGCCATTCT
Cyp7a1	GGGATTGCTGTGGTAGTGAGC	GGTATGGAATCAACCCGTTGTC
Dolk	CAGTGTGGGACCGATACTCCT	CCAAGCAAAGGCATGACCA
Gapdh	CCTGCTTCACCACCTTCTTG	CATGGCCTTCCGTGTTCCTA
Hmgcr	AGCTTGCCCGAATTGTATGTG	TCTGTTGTGAACCATGTGACTTC
Ki67	AGAAGTCCAGGTCTACAG	TCGTTGCTATTGCTAAGG
Mettl3	ATCCAGGCCCATAAGAAACAG	CTATCACTACGGAAGGTTGGG
Rdh16f2	TCTTGGGCAGAGTGTCACTTG	TGCCAGGTATTTCTCTCCATAGA
Slc10a5	CAGCTACCTGCTCGTGAAGTT	AGGTTGACGGTAAAGTCTGTGA

Smpd1	TGGGACTCCTTTGGATGGG	CGGCGCTATGGCACTGAAT
Smpd2	TGGGACATCCCCTACCTGAG	TAGGTGAGCGATAGCCTTTGC
Smpd3	TTCTTCGCCAGCCGCTA	CCACCTGCACCTTGAGAAA
Sptlc1	ACGAGGCTCCAGCATACCAT	TCAGAACGCTCCTGCAACTTG
Sptlc2	AACGGGGAAGTGAGGAACG	CAGCATGGGTGTTTCTTCAAAAG
Srebf1	TGACCCGGCTATTCCGTGA	CTGGGCTGAGCAATACAGTTC
Survivin40	GAGGCTGGCTTCATCCACTG	CTTTTTGCTTGTTGTTGGTCTCC
Triap1	GAGTACGACCAGTGCTTCAAC	CTTGATTGCTTTCTGCACGCA
Xbp1	AGCAGCAAGTGGTGGATTTG	GAGTTTTCTCCCGTAAAAGCTGA

Supplementary Table 12. List of antibodies and suppliers Used for immunoblotting and immunohistochemistry.

Antibody	Cat. No.	Isotype	Suppliers
Mettl3	ab195352	Rabbit	Abcam
Ki67	ab16667	Rabbit	Abcam
Gapdh	10494-1-AP	Rabbit	Proteintech
